# Supplementary material for: Ecosystem-bedrock interaction changes nutrient compartmentalization during early oxidative weathering
Source: Sci Rep. 2019 Oct 18;9:15006. doi: 10.1038/s41598-019-51274-x (PMC6800431; doi:10.1038/s41598-019-51274-x)
Supplement: Supplementary file 1 — Supplementary Info [file 41598_2019_51274_MOESM1_ESM.docx]

Ecosystem-bedrock interaction changes nutrient compartmentalization during early oxidative weathering

Dragos G. Zaharescu^1,2,3^, Carmen I. Burghelea^3^, Katerina Dontsova^3,4^, Jennifer K. Presler^3^, Edward A. Hunt^3^, Kenneth J. Domanik^5^, Mary K. Amistadi^6^, Shana Sandhaus^3,7^, Elise N. Munoz^3,7^, Emily E. Gaddis^3,8^, Miranda Galey^3,9^, María O. Vaquera-Ibarra^3,10^, Maria A. Palacios-Menendez^3,11^, Ricardo Castrejón-Martinez^3,12^, Estefanía C. Roldán-Nicolau^3,12^, Kexin Li^3,13^, Raina M. Maier^4^, Christopher T. Reinhard^1,2^, Jon Chorover^3,4^

^1^Department of Earth and Atmospheric Sciences, Georgia Institute of Technology, Atlanta, GA, U.S.A. ^2^Alternative Earths Team, NASA Astrobiology Institute, University of California, Riverside, CA, U.S.A., ^3^Biosphere 2, The University of Arizona, Tucson, AZ, U.S.A. ^4^Department of Environmental Science, The University of Arizona, Tucson, AZ, U.S.A. ^5^Lunar and Planetary Laboratory, The University of Arizona, Tucson, AZ, U.S.A. ^6^Arizona Laboratory for Emerging Contaminants, The University of Arizona, Tucson, AZ, U.S.A. ^7^Honor’s College, The University of Arizona, Tucson, AZ, U.S.A. ^8^Williams College, Williamstown, MA, U.S.A. ^9^Biology Department, The University of Minnesota, Duluth, MN, U.S.A. ^10^University of the Americas Puebla, Puebla, Mexico. ^11^The University of Caribe, Cancun, Mexico. ^12^National Autonomous University of Mexico. ^13^Department of Computer Sciences, University of Wisconsin-Madison, WI, U.S.A.

Correspondence and requests for materials should be addressed to D.G.Z. (email: zaha_dragos@yahoo.com).

SI 1 Supplementary Methods

**SI 1.1 Experimental setup**

| 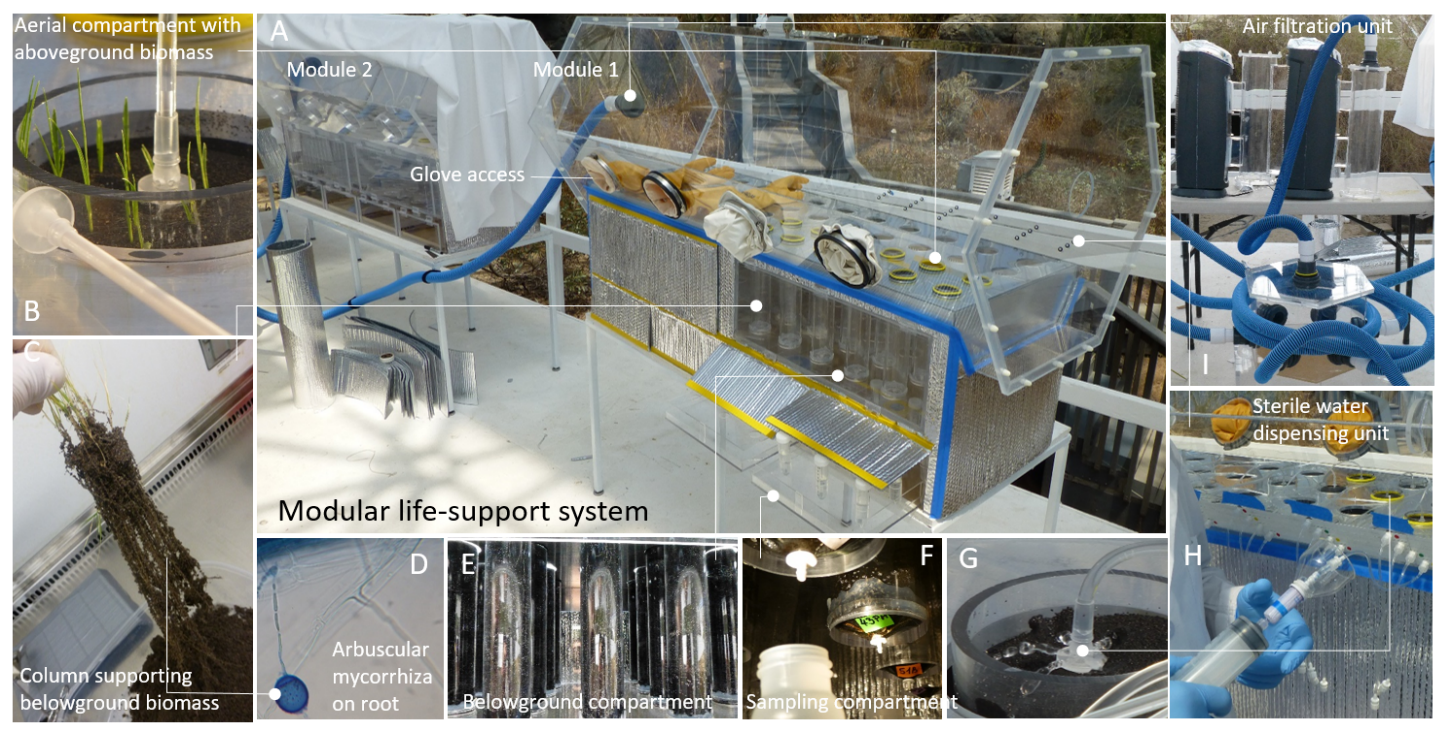 |
| --- |

*Figure s1* **The experimental setup.** Mezocosm chambers represented by six growing units - rockubators (A) in the Desert Biome of Biosphere-2, Arizona. Granular rock - filled columns, 30 x 5 cm long, extend from above - ground (B; Buffalo grass shoots in basalt), to below - ground (C – E; Buffalo grass roots in rhyolite), and to pore water sampling (F) compartments. Purified water and air are delivered through sterile syringe (G and H), and air-purification system (I).

**SI 1.2 Behavior of treatment replicate.** In experimental biogeosciences replicate treatment architecture and associated comparison statistics are required to rule out bias in quantitative results and interpretation, and give predictability power to hypotheses. Treatment triplicates are used as a rule of thumb to establish facts and hence draw conclusions on biogeochemical processes. In systems that are highly sensitive to small variations in processes involved, i.e. high noise, such as our study, the rule-of-three is harder to achieve, hence the reliability of analyzed parameters can be lower. Results from our time series plotting of pore water element content showed that over the studied time a limited number of columns behaved independently of the rest in their triplicate treatment behavior. While they represent real processes, the cause of these unique behaviors has not been identified. To preserve statistical power across the whole experiment, these columns were removed from the general statistical analysis.

SI 2 Supplementary Results

**SI 2.1 Substrate**

| 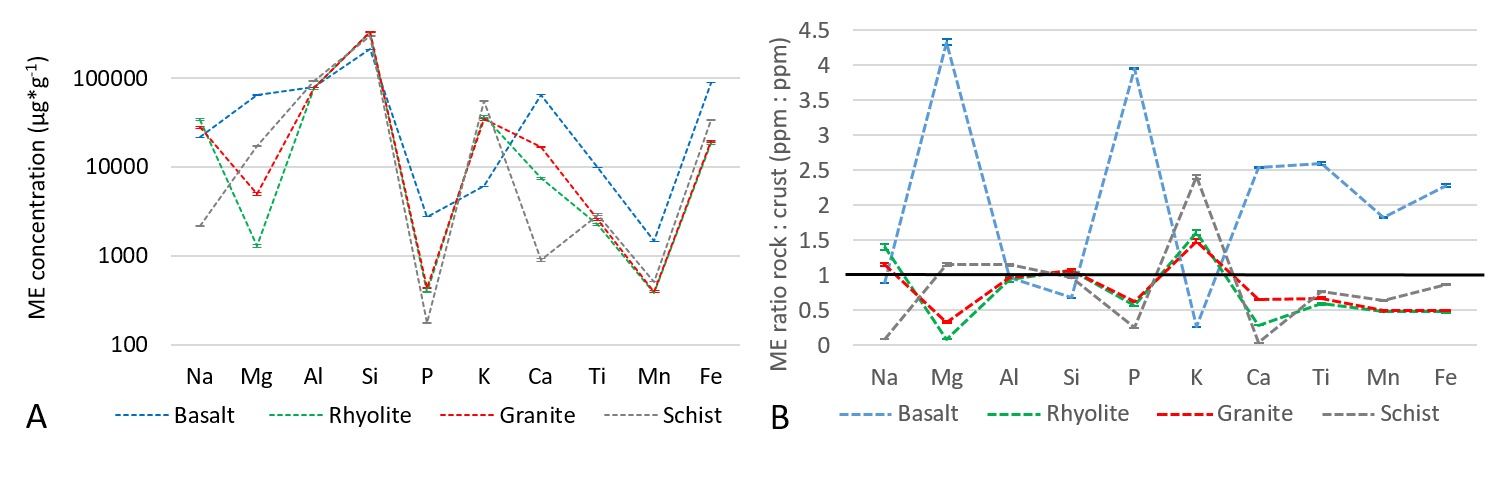 |
| --- |
| *Figure s2* **Substrates elemental composition.** Variability of major element (ME) concentrations in initial rocks (A; log scale), and their values relative to upper continental crust averages^1^, with a reference black line set at unity (B). |

**SI 2.2 Pore water**

| 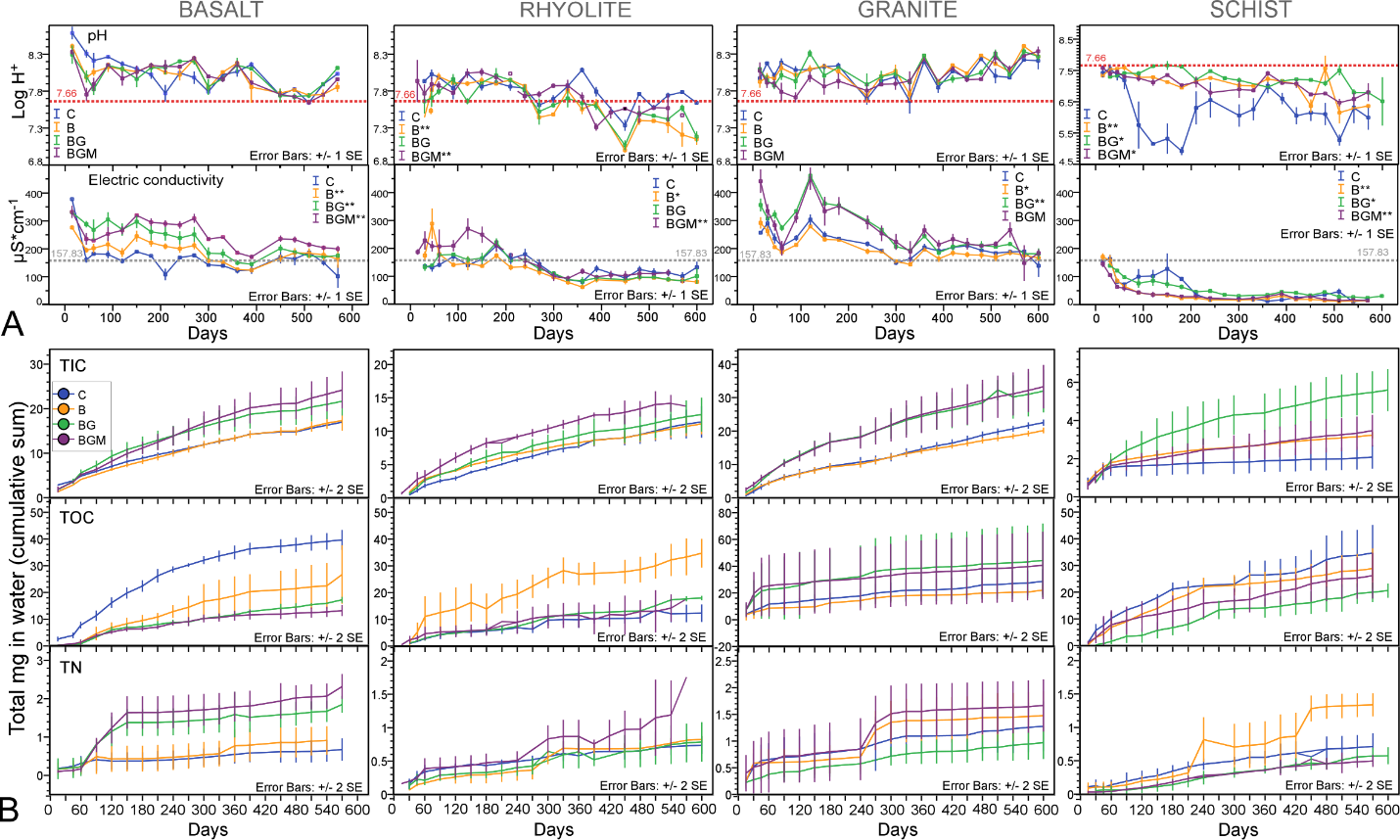 | |  |
| --- | --- | --- |
|  | |  |
| *Figure s3* **Changes in water physico - chemical variates**. Time lapse of (A) pH and electrical conductivity (horizontal dash line is set at the average value for all rocks and treatments), and (B) total anions in pore waters across biotic treatments in each rock. Significance of overall treatment effect in A (legend), was determined at *1SE and **2SE in increasing order of treatment complexity for C-B, B-BG and BG-BGM. A. Biological treatment: C, control; B, microbes; BG, microbes-grass; BGM, microbes-grass-mycorrhiza. |  |  |
| 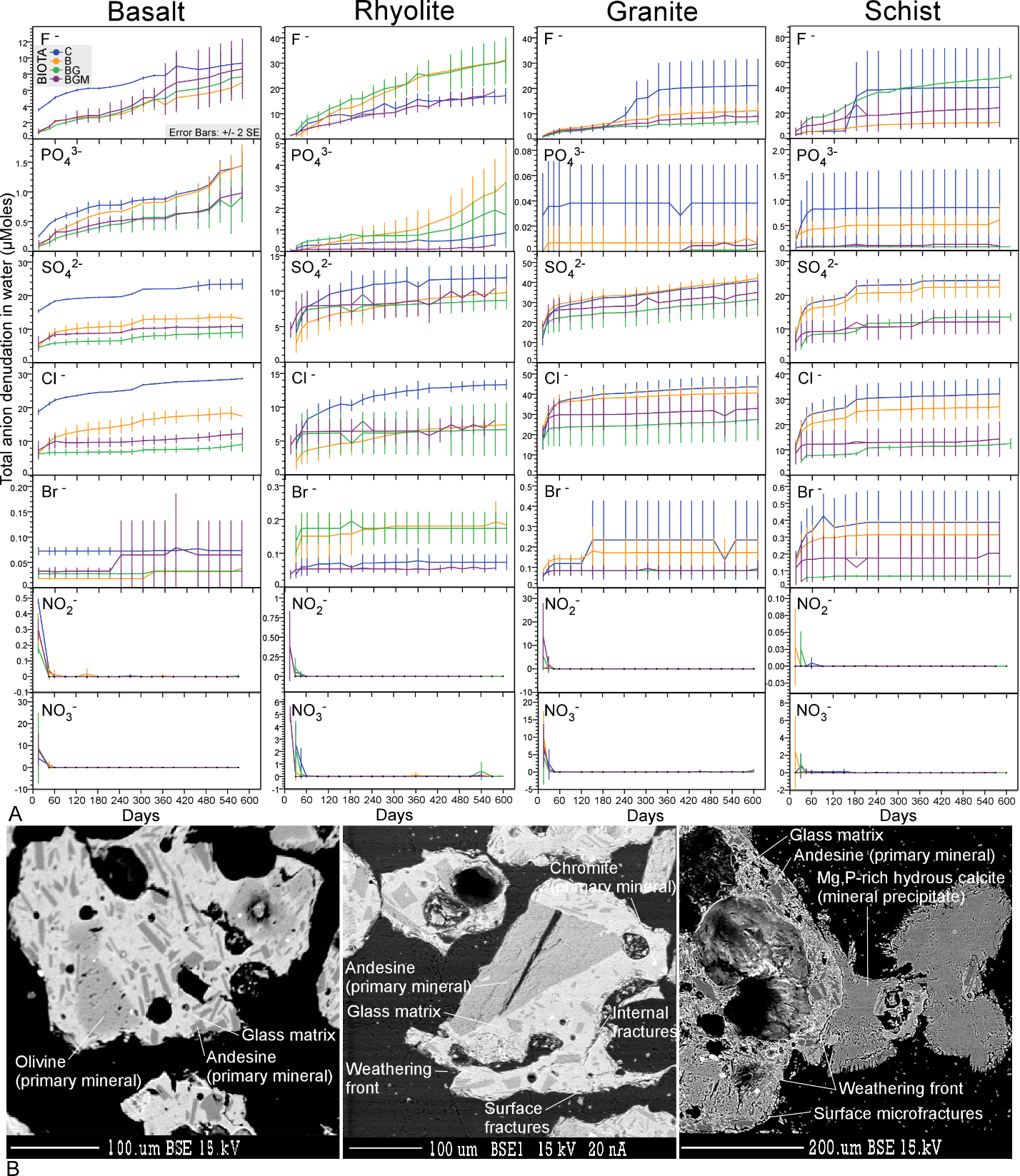 | | |
| *Figure s4* **Anion leaching in time and the physical weathering effect.** (A) Cumulative sum (except nitrite and nitrate) of dissolved anions in pore waters over time, across biotic treatments in each substrate. Legend symbols, for all plots: C, control; B, microbes; BG, microbes-grass; BGM, microbes-grass-mycorrhiza. (B) Electrode microprobe images of basalt grain weathering front at time 0 (left) and end of experiment (center, abiotic control), compared to naturally weathered material from Merriam crater, Arizona (right, with representation of microfractures and precipitated secondary minerals). | | |

| 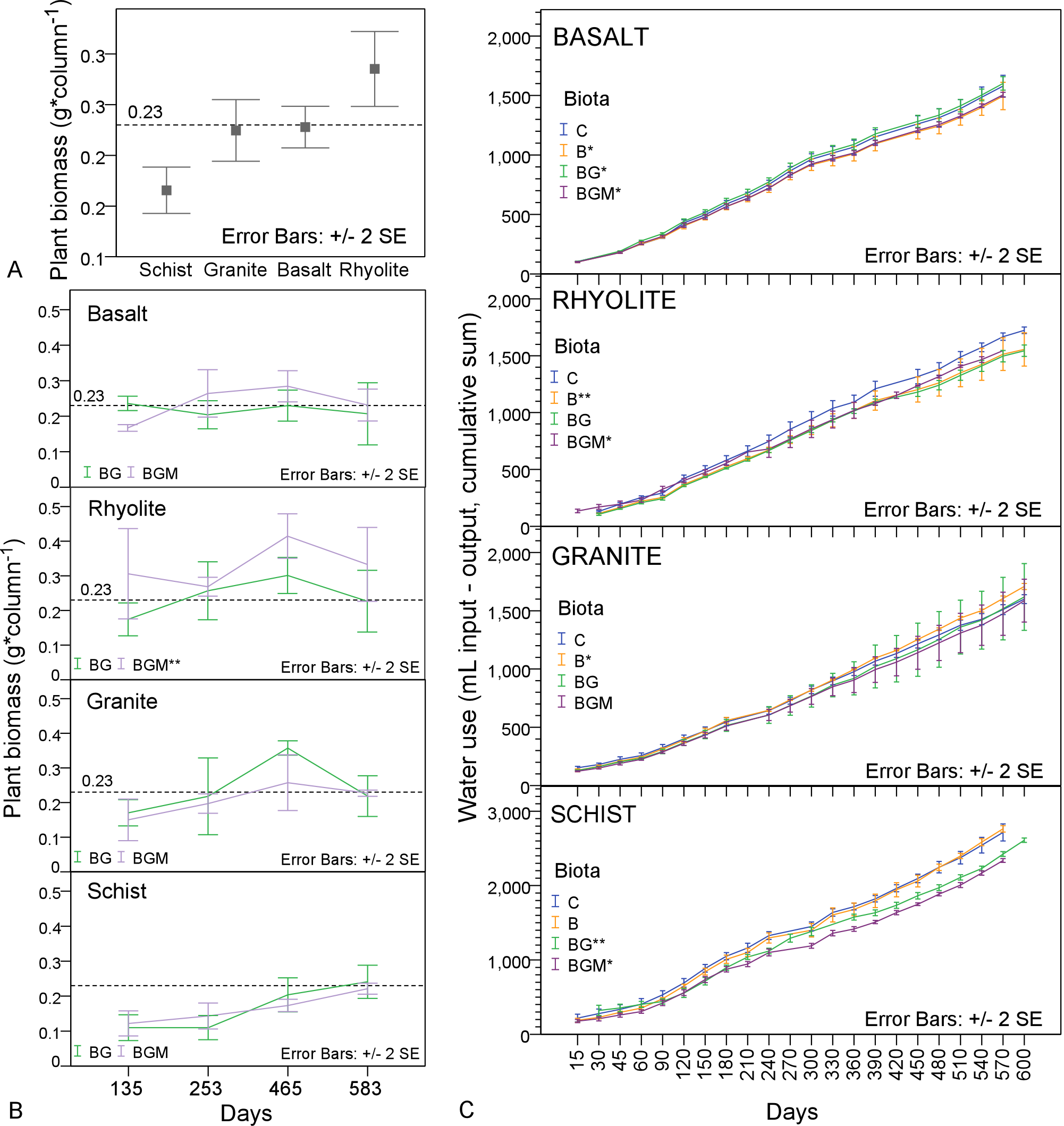 |
| --- |
| *Figure s5* **Changes in plant growth and ecosystem water consumption.** Substrate effect on plant biomass across all four sample events (A) together with time change in plant biomass (B), and total column water consumption (C)- estimated as volume of irrigation input – volume of extracted pore water, over the course of the experiment. Significance of treatment effect displayed in legends of subfigures B and C was determined at *1SE and **2SE, stepwise for C-B, B-BG and BG-BGM. C, control; B, microbes; BG, microbes-grass; BGM, microbes-grass-mycorrhiza. |

*Table s1* **Major pore water physico-chemical parameters.** Descriptives of water consumption, pH, measures of C, N and major ion contents in water leached from four silicate rocks during 20 months biological weathering, across biotic treatments. Except for pH and EC, which show column averages, values are sums per column for the 60 sampling events, and represent total denudation at the end of the experiment. Means and standard errors (SE) were derived from column triplicates. C, control; B, microbial; BG, microbial–grass; and BGM, microbial–grass–arbuscular mycorrhiza.

| Variable | Treatment | Basalt | | | Rhyolite | | | Granite | | | Schist | | |
| --- | --- | --- | --- | --- | --- | --- | --- | --- | --- | --- | --- | --- | --- |
|  |  | Mean ± SE | | | Mean ± SE | | | Mean ± SE | | | Mean ± SE | | |
| Water balance  (input-output; ml) | C | 1579.23 | ± | 45.27 | 1763.58 | ± | 14.25 | 1639.50 | ± | 19.13 | 2754.89 | ± | 57.46 |
|  | B | 1515.66 | ± | 38.88 | 1595.81 | ± | 73.20 | 1748.13 | ± | 13.13 | 2803.11 | ± | 21.52 |
|  | BG | 1601.41 | ± | 28.35 | 1584.14 | ± | 25.17 | 1658.15 | ± | 142.89 | 2650.10 | ± | 14.65 |
|  | BGM | 1506.08 | ± | 10.29 | 1512.08 | ± | 41.03 | 1627.17 | ± | 91.89 | 2377.25 | ± | 13.05 |
| pH | C | 8.03 | ± | 0.013 | 7.80 | ± | 0.026 | 8.04 | ± | 0.018 | 6.27 | ± | 0.23 |
|  | B | 8.01 | ± | 0.0072 | 7.61 | ± | 0.027 | 8.07 | ± | 0.011 | 7.06 | ± | 0.054 |
|  | BG | 8.01 | ± | 0.030 | 7.64 | ± | 0.043 | 8.06 | ± | 0.026 | 7.24 | ± | 0.049 |
|  | BGM | 7.99 | ± | 0.020 | 7.77 | ± | 0.035 | 7.99 | ± | 0.037 | 7.06 | ± | 0.079 |
| EC (µs/cm) | C | 167.09 | ± | 3.16 | 134.30 | ± | 6.56 | 211.59 | ± | 4.85 | 59.23 | ± | 5.75 |
|  | B | 190.49 | ± | 12.89 | 123.89 | ± | 10.38 | 198.93 | ± | 3.20 | 41.92 | ± | 2.14 |
|  | BG | 228.27 | ± | 11.14 | 127.33 | ± | 8.33 | 265.90 | ± | 1.00 | 56.48 | ± | 1.80 |
|  | BGM | 248.05 | ± | 2.52 | 158.32 | ± | 12.01 | 267.70 | ± | 20.43 | 39.75 | ± | 4.81 |
| TC (mg) | C | 56.65 | ± | 1.86 | 23.72 | ± | 2.28 | 51.14 | ± | 4.26 | 36.77 | ± | 5.00 |
|  | B | 40.95 | ± | 4.00 | 45.85 | ± | 3.45 | 42.21 | ± | 1.88 | 32.07 | ± | 3.70 |
|  | BG | 38.93 | ± | 1.47 | 30.45 | ± | 0.84 | 76.34 | ± | 16.95 | 26.19 | ± | 1.76 |
|  | BGM | 37.43 | ± | 1.11 | 28.53 | ± | 2.03 | 73.92 | ± | 15.90 | 29.65 | ± | 3.88 |
| TN (mg) | C | 0.677 | ± | 0.15 | 0.738 | ± | 0.066 | 1.275 | ± | 0.23 | 0.716 | ± | 0.10 |
|  | B | 0.914 | ± | 0.19 | 0.825 | ± | 0.026 | 1.479 | ± | 0.23 | 1.339 | ± | 0.086 |
|  | BG | 1.741 | ± | 0.13 | 0.788 | ± | 0.15 | 0.969 | ± | 0.15 | 0.576 | ± | 0.064 |
|  | BGM | 2.323 | ± | 0.16 | 1.211 | ± | 0.28 | 1.667 | ± | 0.25 | 0.498 | ± | 0.045 |
| Anions (µm) | C | 62.96 | ± | 1.20 | 43.27 | ± | 0.88 | 105.94 | ± | 2.26 | 98.17 | ± | 20.05 |
|  | B | 40.01 | ± | 0.95 | 51.84 | ± | 4.14 | 94.17 | ± | 1.71 | 63.03 | ± | 3.63 |
|  | BG | 26.91 | ± | 1.45 | 48.48 | ± | 2.87 | 66.20 | ± | 9.90 | 75.05 | ± | 0.73 |
|  | BGM | 32.84 | ± | 2.46 | 32.45 | ± | 2.92 | 77.15 | ± | 6.67 | 51.00 | ± | 6.42 |
| Cations (µm) | C | 1436.90 | ± | 10.75 | 983.48 | ± | 80.50 | 1485.22 | ± | 35.28 | 486.16 | ± | 27.71 |
|  | B | 1313.17 | ± | 34.02 | 1168.93 | ± | 15.18 | 1256.16 | ± | 21.87 | 363.38 | ± | 11.99 |
|  | BG | 1438.39 | ± | 79.19 | 1221.83 | ± | 70.35 | 1631.96 | ± | 109.61 | 623.26 | ± | 35.67 |
|  | BGM | 1586.86 | ± | 158.50 | 1012.49 | ± | 16.57 | 1707.76 | ± | 149.20 | 407.73 | ± | 29.49 |

*Table s2.* **Drivers of abiotic dissolution.** Relationship between dissolved elements and principal components (PC) of Principal Component Analysis (PCA; in brackets), together with the predictor variables of PCs as derived from Automatic Linear Model (ALM). ALM accuracy represents total variance explained by predictors, and model values are significant at *p* > 95 %. PCs are on different shades of grey. Anions and predictors were input into the model as molar concentrations, while cations as rock-normalized values (representing preferential loss, *). Input values were averaged across treatment triplicates. PCA rotation method: Varimax with Kaiser normalization.

| **Basalt** | PC (loading) | PC Predictor (% importance) | Model accuracy (%) |  | **Rhyolite** | PC (loading) | PC Predictor (% importance) | Model accuracy (%) |
| --- | --- | --- | --- | --- | --- | --- | --- | --- |
| NO_3_ | 1 (0.99) | Carbonate (54), pH (27), Bicarbonate (19) | 62.2 |  | NO_2_ | 1 (0.95) | H_2_CO_3_ (76), pH (24) | 17.0 |
| SO_4_^2-^ | 1 (0.99) |  |  |  | NO_3_ | 1 (0.95) |  |  |
| Cl- | 1 (0.98) |  |  |  | Br- | 1 (0.92) |  |  |
| NO_2_ | 1 (0.98) |  |  |  | *Fe | 1 (0.92) |  |  |
| Br- | 1 (0.97) |  |  |  | SO_4_^2-^ | 1 (0.89) |  |  |
| *Na | 1 (0.97) |  |  |  | Cl- | 1 (0.89) |  |  |
| F- | 1 (0.96) |  |  |  | PO_4_^3-^ | 1 (0.83) |  |  |
| *Al | 1 (0.95) |  |  |  | *P | 1 (0.75) |  |  |
| *Fe | 1 (0.94) |  |  |  | *Si | 1 (0.72) |  |  |
| *K | 1 (0.90) |  |  |  | *Al | 1 (0.72) |  |  |
| *Si | 1 (0.86) |  |  |  | *Mg | 2 (0.91) | pH (72), Carbonate (28) | 12.0 |
| *Ti | 1 (0.80) |  |  |  | *Ca | 2 (0.91) |  |  |
| *P | 1 (0.76) |  |  |  | *K | 2 (0.81) |  |  |
| PO_4_^3-^ | 1 (0.72) |  |  |  | *Mn | 3 (-0.83) | Carbonate (66), pH (34) | 27.7 |
| *Mg | 2 (0.94) |  |  |  | F- | 3 (0.74) |  |  |
| *Ca | 2 (0.90) |  |  |  | *Na | 3 (0.65) |  |  |
| *Mn | 3 (0.91) | TOC (-,100) | 44.6 |  | *Ti | 4 (0.96) |  |  |
| Total variance explained (%): PC1 (70.2), PC2 (12), PC3 (8). | | | |  | Total variance explained (%): PC1 (46), PC2 (17.6), PC3 (15), PC4 (7.9). | | | |

| **Granite** | PC (loading) | PC Predictor (% importance) | Model accuracy (%) |  | **Schist** | PC (loading) | PC Predictor (% importance) | Model accuracy (%) |
| --- | --- | --- | --- | --- | --- | --- | --- | --- |
| NO_2_ | 1 (0.97) | TOC (100) | 47.40 |  | *Na | 1 (0.99) | Carbonate (42), Bicarbonate (41), pH (9), TOC (5), H_2_CO_3_ (3) | 97.0 |
| Cl- | 1 (0.96) |  |  |  | *P | 1 (0.98) |  |  |
| SO_4_^2-^ | 1 (0.96) |  |  |  | Cl- | 1 (0.96) |  |  |
| NO_3_ | 1 (0.93) |  |  |  | Br- | 1 (0.95) |  |  |
| PO_4_^3-^ | 1 (0.88) |  |  |  | SO_4_^2-^ | 1 (0.94) |  |  |
| *Na | 1 (0.83) |  |  |  | PO_4_^3-^ | 1 (0.93) |  |  |
| *Si | 1 (0.81) |  |  |  | *Si | 1 (0.73) |  |  |
| *K | 2 (0.94) |  |  |  | *Ca | 1 (0.71) |  |  |
| *Ca | 2 (0.88) |  |  |  | NO_3_ | 1 (0.65) |  |  |
| *Fe | 2 (0.67) |  |  |  | *Mn | 2 (0.93) | pH (56), TOC (30), H_2_CO_3_ (14) | 84.0 |
| Br- | 2 (-0.51) |  |  |  | *Mg | 2 (0.83) |  |  |
| *Al | 3 (-0.90) | TOC(54), Bicarbonate (46) | 47.70 |  | F- | 2 (0.80) |  |  |
| *Mg | 3 (0.75) |  |  |  | *K | 2 (0.78) |  |  |
| *P | 3 (0.71) |  |  |  | *Fe | 3 (0.89) |  |  |
| *Mn | 3 (-0.60) |  |  |  | *Ti | 3 (0.79) |  |  |
| *Ti | 4 (0.93) |  |  |  | *Al | 3 (0.75) |  |  |
| F- | 5 (0.95) | H_2_CO_3_(68), pH(32) | 32.10 |  | NO_2_ | 4 (0.90) |  |  |
| Total variance explained (%): PC1 (38), PC2 (19.3), PC3 (15.8), PC4 (8), PC5 (7.8). | | | |  | Total variance explained (%): PC1 (44.9), PC2 (21.4), PC3 (13.4), PC4 (7.5). | | | |

| \| 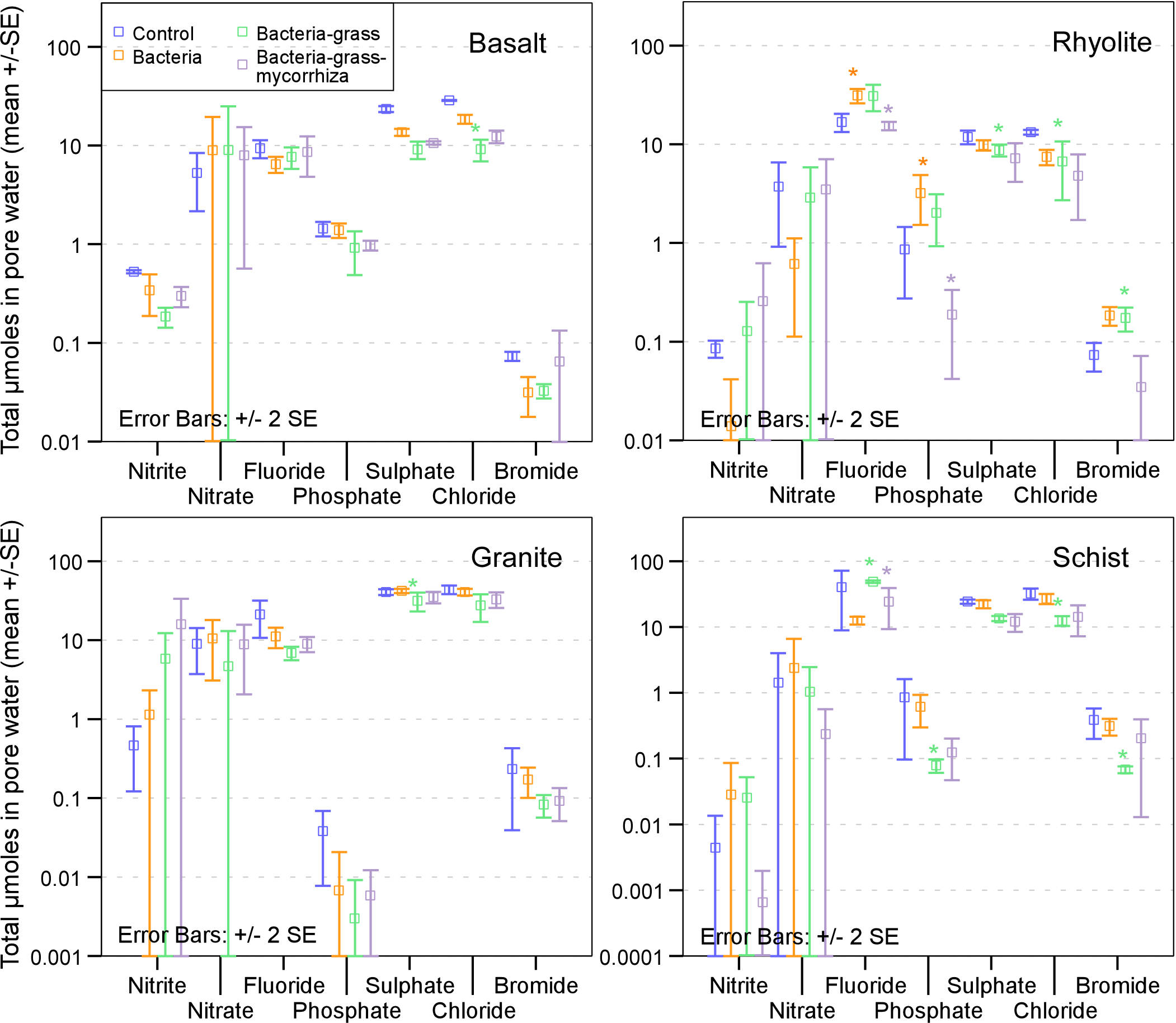 \| \| --- \| \| *Figure s6* **Biotic effect on pore water anions export.** Variability in total anions (mass in water summed over the 60 sampling events) among biotic tratments in the four rocks. Means and standard errors are calculated from column triplicates. Y axis is on logarithmic scale. Large nitrite and nitrate SEs are due to high peak values measured the first two months. \| |
| --- | --- | --- |

*Table s3.* **Drivers of biotic dissolution (a).** Grouping of dissolved elements by Principal Component Analysis, their correlation coefficient to the group (PC loading, in brackets), % total variance explained by each PC (table bottom), and relative importance (%) of PC predictors, derived from Automatic Linear Model (ALM). Total variance explained by predictors (%) is significant at *p* > 95 %. Anions and predictors (pH, H^+^, H_2_CO_3_, carbonate, bicarbonate, total organic carbon-TOC) were input into the model as molar concentrations, while cations as rock-normalized values (representing preferential loss, *). Input values were averaged across treatment triplicates. PCA rotation method: Varimax with Kaiser normalization. Treatments: B, microbial; BG, microbial–grass; and BGM, microbial–grass–arbuscular mycorrhiza.

|  |  |
| --- | --- |
|  |  |

*Figure s7.* **Drivers of biotic dissolution (b).** Relative contribution of each driver to the dissolution of elements (% total variance explained by driver out of total explained by PCA; Table *s3*), derived from Principal Component analysis (PCA) and Automatic Linear Model (ALM). Values are significant at *p* > 95 %. Treatments: B, microbial; BG, microbial–grass; and BGM, microbial–grass–arbuscular mycorrhiza.

**SI 2.3 Vascular plant**

| 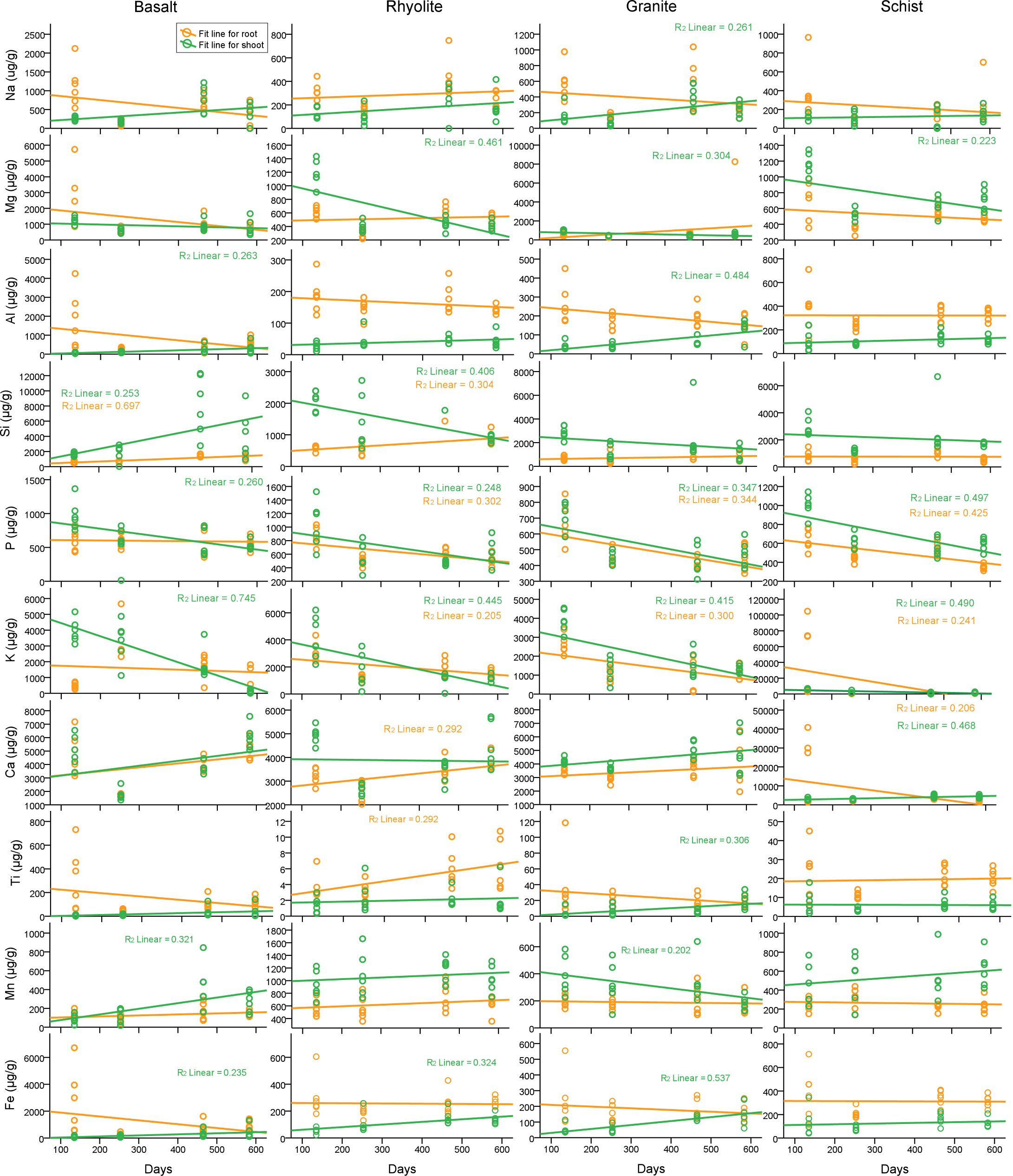 |
| --- |
| *Figure s8* **Major element content during plant development.** Time changes in major element concentrations (ug/dry biomass) in plants grown in four silicate rocks, together with their fit lines. Only r^2^ > 0.2 is displayed. |

| 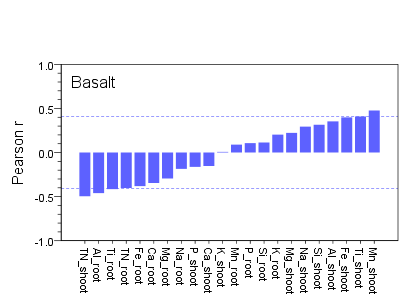 | 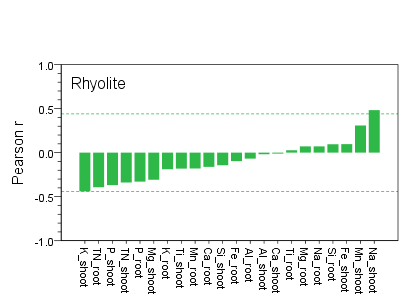 |
| --- | --- |
| 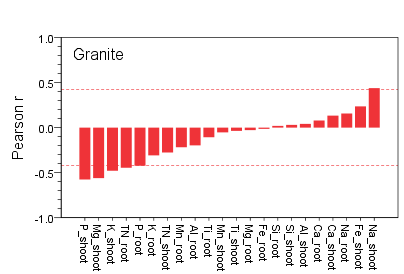 | 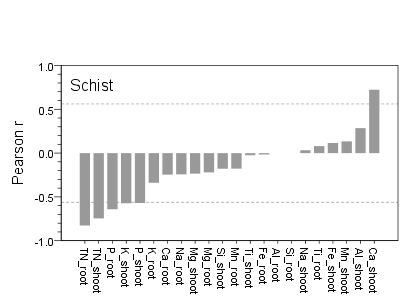 |
| *Figure s9* **Factors limiting plant development.** Relationship between plant biomass and its element concentrations, with values above and below dash lines significant at 95% level. | |

**SI 2.4 Biological Signature index**

*Table s4.* **Abiotic component (Z) of Biological Signature Index.** Rescaled (0-100) fractions moles element to moles total cations (summed across all sampling events) in extract : parent rock, averaged across column triplicates.

|  | Water | | | | | Exchangeable | | | | | | Poorly crystalline | | | |
| --- | --- | --- | --- | --- | --- | --- | --- | --- | --- | --- | --- | --- | --- | --- | --- |
| Element | | Basalt | Rhyolite | Granite | Schist | | Basalt | Rhyolite | Granite | Schist | Basalt | | Rhyolite | Granite | Schist |
| Na | | 85.14 | 9.84 | 54.20 | 42.86 | | 56.30 | 2.27 | 14.75 | 69.78 | 15.15 | | 51.45 | 31.16 | 38.53 |
| Mg | | 5.88 | 70.95 | 67.21 | 79.22 | | 21.84 | 12.84 | 37.10 | 43.26 | 18.89 | | 73.83 | 76.31 | 1.13 |
| Al | | 87.79 | 24.69 | 35.85 | 75.76 | | 43.56 | 17.00 | 8.13 | 34.42 | 62.99 | | 20.05 | 49.45 | 6.47 |
| Si | | 88.90 | 66.17 | 57.12 | 9.86 | | 45.58 | 63.37 | 34.07 | 83.39 | 79.04 | | 42.28 | 27.20 | 19.88 |
| P | | 66.82 | 19.40 | 28.85 | 53.06 | | 53.12 | 86.11 | 52.30 | 24.24 | 9.08 | | 16.73 | 69.23 | 46.64 |
| K | | 66.88 | 29.04 | 39.90 | 20.94 | | 82.47 | 15.35 | 21.66 | 8.04 | 32.63 | | 58.40 | 35.64 | 89.34 |
| Ca | | 11.28 | 79.92 | 45.37 | 93.25 | | 47.42 | 89.50 | 75.56 | 40.05 | 33.14 | | 39.97 | 51.14 | 63.81 |
| Ti | | 37.59 | 29.43 | 28.99 | 21.63 | | 23.01 | 19.70 | 41.51 | 26.33 | 21.50 | | 12.17 | 48.96 | 16.68 |
| Mn | | 9.79 | 38.99 | 63.02 | 74.92 | | 54.75 | 50.78 | 26.84 | 59.35 | 12.79 | | 85.00 | 0.00 | 33.33 |
| Fe | | 22.79 | 31.93 | 42.04 | 60.95 | | 24.16 | 13.42 | 7.30 | 18.54 | 75.06 | | 54.90 | 64.02 | 10.98 |

**SI 2.5 Global projections**

*Table s5.* **Global denudation estimates.** Water denudation of major rock cations, estimated in 10^10^ moles/year, from four igneous lithologies under different ecosystem scenarios: C, abiotic; B, microbial; BG, microbial-vascular plant; and BGM, microbial- plant- arbuscular mycorrhiza.

| Element | Biota | Basalt | Rhyolite | Granite | Schist |
| --- | --- | --- | --- | --- | --- |
| Na | C | 73.9 | 8.64 | 27.9 | 75.5 |
|  | B | 45.2 | 18.2 | 33.2 | 116 |
|  | BG | 39.9 | 17.2 | 21.3 | 91.5 |
|  | BGM | 36.3 | 8.51 | 21.9 | 90.3 |
| Mg | C | 4.69 | 2.82 | 7.47 | 19.5 |
|  | B | 7.38 | 3.00 | 8.65 | 17.6 |
|  | BG | 7.19 | 2.65 | 6.19 | 21.3 |
|  | BGM | 6.75 | 2.97 | 6.86 | 18.5 |
| Al | C | 0.150 | 0.00868 | 0.0403 | 0.863 |
|  | B | 0.0843 | 0.0125 | 0.0569 | 0.775 |
|  | BG | 0.0694 | 0.0230 | 0.0283 | 0.467 |
|  | BGM | 0.0460 | 0.00405 | 0.0283 | 0.757 |
| Si | C | 24.1 | 13.4 | 19.2 | 65.3 |
|  | B | 17.0 | 20.6 | 23.9 | 96.8 |
|  | BG | 13.5 | 20.8 | 13.5 | 124 |
|  | BGM | 12.4 | 10.8 | 14.4 | 109 |
| P | C | 0.413 | 0.118 | 0.147 | 1.68 |
|  | B | 0.320 | 0.341 | 0.217 | 3.04 |
|  | BG | 0.239 | 0.290 | 0.139 | 0.893 |
|  | BGM | 0.178 | 0.0916 | 0.184 | 1.95 |
| K | C | 3.52 | 1.25 | 7.52 | 31.2 |
|  | B | 2.60 | 2.02 | 8.54 | 37.7 |
|  | BG | 2.31 | 2.12 | 7.20 | 54.7 |
|  | BGM | 2.10 | 1.37 | 7.17 | 51.3 |
| Ca | C | 33.6 | 12.53 | 90.7 | 78.7 |
|  | B | 30.9 | 12.3 | 89.5 | 80.6 |
|  | BG | 31.1 | 12.7 | 92.0 | 76.8 |
|  | BGM | 31.5 | 12.4 | 91.3 | 79.6 |
| Ti | C | 0.00102 | 0.000234 | 0.000412 | 0.0113 |
|  | B | 0.000630 | 0.000518 | 0.000510 | 0.0172 |
|  | BG | 0.000905 | 0.000580 | 0.000456 | 0.0130 |
|  | BGM | 0.000535 | 0.000129 | 0.000374 | 0.0219 |
| Mn | C | 0.00745 | 0.139 | 0.0577 | 0.743 |
|  | B | 0.0120 | 0.0586 | 0.0383 | 0.175 |
|  | BG | 0.00708 | 0.0891 | 0.0437 | 0.0410 |
|  | BGM | 0.0234 | 0.0619 | 0.0450 | 0.0880 |
| Fe | C | 0.00632 | 0.00328 | 0.00722 | 0.204 |
|  | B | 0.00395 | 0.00388 | 0.00649 | 0.250 |
|  | BG | 0.00483 | 0.00692 | 0.00764 | 0.136 |
|  | BGM | 0.00654 | 0.00145 | 0.00687 | 0.256 |
| **Total**  **cations** | **C** | **140.4** | **38.9** | **153** | **273.7** |
|  | **B** | **103.5** | **56.5** | **164.1** | **353** |
|  | **BG** | **94.3** | **55.9** | **140.4** | **369.9** |
|  | **BGM** | **89.3** | **36.2** | **141.9** | **351.8** |

The contribution of abiotic and biotic processes to global denudation of terrestrial surface was calculated by normalizing total moles of element extracted during the whole experiment to global Ca + Mg estimated from large river data^2^, using the equation E. *s1*. The term *i_G_* represents the global (*G*) value of element (*i)* of interest (moles * year^-1^), and *i_E_* is total moles element in water from experiment *E*. Values were adjusted (multiplied) to the relative contribution (%) of the four rocks to the global lithology^3^.

(E. *s1*)

$i_{G}= \frac{i_{E}}{{(Ca+Mg)}_{E}} {(Ca+Mg)}_{G}$

The contribution of different ecosystem components to global denudation was estimated from their ratio in the experiment in increasing order of complexity: GBM : GB : B : C. Results are in Table *s5.*

SI References

1. Rudnick, R. & Gao, S. Composition of the continental crust. *Treatise on Geochemistry* **3,** 1–64 (2003).

2. Gaillardet, J., Duprre, B., Louvat, P. & Allegre, C. J. Global silicate weathering and CO2 consumption rates deduced from the chemistry of large rivers. *Chem. Geol.* **159,** 3–30 (1999).

3. Suchet, P. A. Worldwide distribution of continental rock lithology: Implications for the atmospheric/soil CO 2 uptake by continental weathering and alkalinity river transport to the oceans. *Global Biogeochem. Cycles* **17,** (2003).
