# Supplementary material for: Ecosystem-bedrock interaction changes nutrient compartmentalization during early oxidative weathering
Source: Sci Rep. 2019 Oct 18;9:15006. doi: 10.1038/s41598-019-51274-x (PMC6800431; doi:10.1038/s41598-019-51274-x)
Supplement: Supplementary file 2 — Dataset 1 [file 41598_2019_51274_MOESM2_ESM.docx]

Ecosystem-bedrock interaction changes nutrient compartmentalization during early oxidative weathering

Dragos G. Zaharescu^1,2,3^, Carmen I. Burghelea^3^, Katerina Dontsova^3,4^, Jennifer K. Presler^3^, Edward A. Hunt^3^, Kenneth J. Domanik^5^, Mary K. Amistadi^6^, Shana Sandhaus^3,7^, Elise N. Munoz^3,7^, Emily E. Gaddis^3,8^, Miranda Galey^3,9^, María O. Vaquera-Ibarra^3,10^, Maria A. Palacios-Menendez^3,11^, Ricardo Castrejón-Martinez^3,12^, Estefanía C. Roldán-Nicolau^3,12^, Kexin Li^3,13^, Raina M. Maier^4^, Christopher T. Reinhard^1,2^, Jon Chorover^3,4^

^1^Department of Earth and Atmospheric Sciences, Georgia Institute of Technology, Atlanta, GA, U.S.A. ^2^Alternative Earths Team, NASA Astrobiology Institute, University of California, Riverside, CA, U.S.A., ^3^Biosphere 2, The University of Arizona, Tucson, AZ, U.S.A. ^4^Department of Environmental Science, The University of Arizona, Tucson, AZ, U.S.A. ^5^Lunar and Planetary Laboratory, The University of Arizona, Tucson, AZ, U.S.A. ^6^Arizona Laboratory for Emerging Contaminants, The University of Arizona, Tucson, AZ, U.S.A. ^7^Honor’s College, The University of Arizona, Tucson, AZ, U.S.A. ^8^Williams College, Williamstown, MA, U.S.A. ^9^Biology Department, The University of Minnesota, Duluth, MN, U.S.A. ^10^University of the Americas Puebla, Puebla, Mexico. ^11^The University of Caribe, Cancun, Mexico. ^12^ National Autonomous University of Mexico. ^13^Department of Computer Sciences, University of Wisconsin-Madison, WI, U.S.A.

Correspondence and requests for materials should be addressed to D.G.Z. (zaha_dragos@yahoo.com).

*Dataset 1* **A mass balance of abiotic and biological weathering.** Compartmentalization of major elements into shoot, root, pore water, exchangeable (ammonium acetate extract; aa), poorly crystalline (ammonium oxalate extract; ao), and unreacted rock, following a two – year weathering experiment of basalt, rhyolite, granite and schist. T0, unreacted rock; C, abiotic control; B, microbes; BG, microbes-grass; BGM, microbes-grass-arbuscular mycorrhiza. Reacted treatments are highlighted in color.

|  |  |  |  |  |  |  |  | **T0** |  |  |  | **C** |  |  |  | **B** |  |  |  | **BG** |  |  |  | **BGM** |  |  |
| --- | --- | --- | --- | --- | --- | --- | --- | --- | --- | --- | --- | --- | --- | --- | --- | --- | --- | --- | --- | --- | --- | --- | --- | --- | --- | --- |
| Index | Elem Code | Rock Code | Fraction Code | Element | ROCK | Fraction | N | Mean % | ± | SE | N | Mean % | ± | Std. Error | N | Mean % | ± | Std. Error | N | Mean % | ± | Std. Error | N | Mean % | ± | Std. Error |
| 1 | 1 | 1 | 1 | Na | Basalt | shoot |  |  |  |  |  |  |  |  |  |  |  |  | 3 | 0.00018 | ± | 0.00018 | 3 | 0.00027 | ± | 0.00007 |
| 2 | 1 | 1 | 2 | Na | Basalt | root |  |  |  |  |  |  |  |  |  |  |  |  | 3 | 0.00026 | ± | 0.00026 | 3 | 0.00034 | ± | 0.00014 |
| 3 | 1 | 1 | 3 | Na | Basalt | water |  |  |  |  | 3 | 0.11325 | ± | 0.00235 | 3 | 0.08752 | ± | 0.00046 | 3 | 0.09211 | ± | 0.00019 | 3 | 0.09778 | ± | 0.00683 |
| 4 | 1 | 1 | 4 | Na | Basalt | aa | 3 | 0.12844 | ± | 0.00164 | 3 | 0.08226 | ± | 0.00542 | 3 | 0.07701 | ± | 0.00192 | 3 | 0.08256 | ± | 0.00536 | 3 | 0.07346 | ± | 0.00179 |
| 5 | 1 | 1 | 5 | Na | Basalt | ao | 3 | 4.86919 | ± | 0.06573 | 3 | 2.16176 | ± | 0.10964 | 3 | 2.41865 | ± | 0.09938 | 3 | 2.30252 | ± | 0.01540 | 3 | 2.57018 | ± | 0.17745 |
| 6 | 1 | 1 | 6 | Na | Basalt | unextracted | 3 | 95.00238 | ± | 0.06554 | 3 | 97.64273 | ± | 0.11348 | 3 | 97.41683 | ± | 0.09769 | 3 | 97.52237 | ± | 0.01025 | 3 | 97.25796 | ± | 0.18218 |
| 7 | 1 | 2 | 1 | Na | Rhyolite | shoot |  |  |  |  |  |  |  |  |  |  |  |  | 3 | 0.00006 | ± | 0.00003 | 3 | 0.00014 | ± | 0.00004 |
| 8 | 1 | 2 | 2 | Na | Rhyolite | root |  |  |  |  |  |  |  |  |  |  |  |  | 3 | 0.00013 | ± | 0.00005 | 3 | 0.00020 | ± | 0.00003 |
| 9 | 1 | 2 | 3 | Na | Rhyolite | water |  |  |  |  | 3 | 0.02099 | ± | 0.00166 | 3 | 0.03717 | ± | 0.00062 | 3 | 0.03738 | ± | 0.00266 | 3 | 0.02384 | ± | 0.00114 |
| 10 | 1 | 2 | 4 | Na | Rhyolite | aa | 3 | 0.04535 | ± | 0.00084 | 3 | 0.02500 | ± | 0.00048 | 3 | 0.04175 | ± | 0.00384 | 3 | 0.05282 | ± | 0.01038 | 3 | 0.02419 | ± | 0.00103 |
| 11 | 1 | 2 | 5 | Na | Rhyolite | ao | 3 | 0.00884 | ± | 0.00030 | 3 | 0.03410 | ± | 0.00650 | 3 | 0.02470 | ± | 0.00381 | 3 | 0.02425 | ± | 0.00039 | 3 | 0.01755 | ± | 0.00076 |
| 12 | 1 | 2 | 6 | Na | Rhyolite | unextracted | 3 | 99.94581 | ± | 0.00111 | 3 | 99.91991 | ± | 0.00532 | 3 | 99.89637 | ± | 0.00209 | 3 | 99.88536 | ± | 0.01254 | 3 | 99.93407 | ± | 0.00219 |
| 13 | 1 | 3 | 1 | Na | Granite | shoot |  |  |  |  |  |  |  |  |  |  |  |  | 3 | 0.00012 | ± | 0.00001 | 3 | 0.00012 | ± | 0.00003 |
| 14 | 1 | 3 | 2 | Na | Granite | root |  |  |  |  |  |  |  |  |  |  |  |  | 3 | 0.00018 | ± | 0.00005 | 3 | 0.00019 | ± | 0.00001 |
| 15 | 1 | 3 | 3 | Na | Granite | water |  |  |  |  | 3 | 0.03338 | ± | 0.00085 | 3 | 0.03079 | ± | 0.00122 | 3 | 0.03070 | ± | 0.00112 | 3 | 0.03418 | ± | 0.00156 |
| 16 | 1 | 3 | 4 | Na | Granite | aa | 3 | 0.05560 | ± | 0.00069 | 3 | 0.01184 | ± | 0.00127 | 3 | 0.01183 | ± | 0.00077 | 3 | 0.01258 | ± | 0.00252 | 3 | 0.01734 | ± | 0.00243 |
| 17 | 1 | 3 | 5 | Na | Granite | ao | 3 | 0.02427 | ± | 0.00077 | 3 | 0.02325 | ± | 0.00078 | 3 | 0.02087 | ± | 0.00116 | 3 | 0.02309 | ± | 0.00134 | 3 | 0.03723 | ± | 0.00248 |
| 18 | 1 | 3 | 6 | Na | Granite | unextracted | 3 | 99.92012 | ± | 0.00038 | 3 | 99.93152 | ± | 0.00050 | 3 | 99.93651 | ± | 0.00153 | 3 | 99.93334 | ± | 0.00164 | 3 | 99.91093 | ± | 0.00487 |
| 19 | 1 | 4 | 1 | Na | Schist | shoot |  |  |  |  |  |  |  |  |  |  |  |  | 3 | 0.00092 | ± | 0.00034 | 3 | 0.00134 | ± | 0.00024 |
| 20 | 1 | 4 | 2 | Na | Schist | root |  |  |  |  |  |  |  |  |  |  |  |  | 3 | 0.00210 | ± | 0.00089 | 3 | 0.00436 | ± | 0.00209 |
| 21 | 1 | 4 | 3 | Na | Schist | water |  |  |  |  | 3 | 0.24088 | ± | 0.01752 | 3 | 0.21728 | ± | 0.00376 | 3 | 0.31277 | ± | 0.01020 | 3 | 0.22695 | ± | 0.01283 |
| 22 | 1 | 4 | 4 | Na | Schist | aa | 3 | 0.38854 | ± | 0.01084 | 3 | 0.06822 | ± | 0.00955 | 3 | 0.06038 | ± | 0.01441 | 3 | 0.07227 | ± | 0.00416 | 3 | 0.03843 | ± | 0.00149 |
| 23 | 1 | 4 | 5 | Na | Schist | ao | 3 | 0.02927 | ± | 0.00953 | 3 | 0.16037 | ± | 0.02824 | 3 | 0.19489 | ± | 0.01331 | 3 | 0.19972 | ± | 0.01410 | 3 | 0.16741 | ± | 0.00860 |
| 24 | 1 | 4 | 6 | Na | Schist | unextracted | 3 | 99.58219 | ± | 0.01193 | 3 | 99.53053 | ± | 0.02892 | 3 | 99.52745 | ± | 0.00490 | 3 | 99.41222 | ± | 0.00890 | 3 | 99.56150 | ± | 0.01628 |
| 25 | 2 | 1 | 1 | Mg | Basalt | shoot |  |  |  |  |  |  |  |  |  |  |  |  | 3 | 0.00010 | ± | 0.00004 | 3 | 0.00022 | ± | 0.00009 |
| 26 | 2 | 1 | 2 | Mg | Basalt | root |  |  |  |  |  |  |  |  |  |  |  |  | 3 | 0.00020 | ± | 0.00006 | 3 | 0.00032 | ± | 0.00001 |
| 27 | 2 | 1 | 3 | Mg | Basalt | water |  |  |  |  | 3 | 0.00252 | ± | 0.00012 | 3 | 0.00501 | ± | 0.00020 | 3 | 0.00581 | ± | 0.00038 | 3 | 0.00637 | ± | 0.00069 |
| 28 | 2 | 1 | 4 | Mg | Basalt | aa | 3 | 0.05596 | ± | 0.00084 | 3 | 0.04114 | ± | 0.00069 | 3 | 0.04658 | ± | 0.00163 | 3 | 0.04610 | ± | 0.00262 | 3 | 0.04543 | ± | 0.00240 |
| 29 | 2 | 1 | 5 | Mg | Basalt | ao | 3 | 2.45053 | ± | 0.01412 | 3 | 1.34080 | ± | 0.06703 | 3 | 1.47781 | ± | 0.05354 | 3 | 1.40765 | ± | 0.00800 | 3 | 1.56282 | ± | 0.08980 |
| 30 | 2 | 1 | 6 | Mg | Basalt | unextracted | 3 | 97.49351 | ± | 0.01364 | 3 | 98.61554 | ± | 0.06756 | 3 | 98.47059 | ± | 0.05339 | 3 | 98.54014 | ± | 0.00690 | 3 | 98.38483 | ± | 0.09051 |
| 31 | 2 | 2 | 1 | Mg | Rhyolite | shoot |  |  |  |  |  |  |  |  |  |  |  |  | 3 | 0.00460 | ± | 0.00119 | 3 | 0.00727 | ± | 0.00137 |
| 32 | 2 | 2 | 2 | Mg | Rhyolite | root |  |  |  |  |  |  |  |  |  |  |  |  | 3 | 0.00663 | ± | 0.00138 | 3 | 0.01173 | ± | 0.00229 |
| 33 | 2 | 2 | 3 | Mg | Rhyolite | water |  |  |  |  | 3 | 0.19012 | ± | 0.02575 | 3 | 0.17099 | ± | 0.02302 | 3 | 0.15907 | ± | 0.02735 | 3 | 0.23078 | ± | 0.00965 |
| 34 | 2 | 2 | 4 | Mg | Rhyolite | aa | 3 | 6.15617 | ± | 0.19402 | 3 | 5.62224 | ± | 0.11237 | 3 | 6.63579 | ± | 0.07866 | 3 | 6.29286 | ± | 0.16976 | 3 | 5.54934 | ± | 0.07184 |
| 35 | 2 | 2 | 5 | Mg | Rhyolite | ao | 3 | 1.34817 | ± | 0.04163 | 3 | 1.41582 | ± | 0.06029 | 3 | 1.20626 | ± | 0.02766 | 3 | 1.21219 | ± | 0.01538 | 3 | 1.24135 | ± | 0.03234 |
| 36 | 2 | 2 | 6 | Mg | Rhyolite | unextracted | 3 | 92.49566 | ± | 0.22592 | 3 | 92.77183 | ± | 0.13302 | 3 | 91.98697 | ± | 0.10224 | 3 | 92.32465 | ± | 0.16132 | 3 | 92.95953 | ± | 0.10267 |
| 37 | 2 | 3 | 1 | Mg | Granite | shoot |  |  |  |  |  |  |  |  |  |  |  |  | 3 | 0.00130 | ± | 0.00012 | 3 | 0.00171 | ± | 0.00021 |
| 38 | 2 | 3 | 2 | Mg | Granite | root |  |  |  |  |  |  |  |  |  |  |  |  | 3 | 0.01225 | ± | 0.01019 | 3 | 0.00226 | ± | 0.00014 |
| 39 | 2 | 3 | 3 | Mg | Granite | water |  |  |  |  | 3 | 0.05373 | ± | 0.00098 | 3 | 0.04818 | ± | 0.00231 | 3 | 0.05354 | ± | 0.00423 | 3 | 0.06439 | ± | 0.00622 |
| 40 | 2 | 3 | 4 | Mg | Granite | aa | 3 | 0.38638 | ± | 0.00447 | 3 | 0.15777 | ± | 0.00838 | 3 | 0.17141 | ± | 0.02231 | 3 | 0.12463 | ± | 0.01057 | 3 | 0.13064 | ± | 0.00476 |
| 41 | 2 | 3 | 5 | Mg | Granite | ao | 3 | 0.73396 | ± | 0.04131 | 3 | 0.13401 | ± | 0.00970 | 3 | 0.12042 | ± | 0.00824 | 3 | 0.12984 | ± | 0.00457 | 3 | 0.14108 | ± | 0.01102 |
| 42 | 2 | 3 | 6 | Mg | Granite | unextracted | 3 | 98.87965 | ± | 0.04546 | 3 | 99.65449 | ± | 0.01445 | 3 | 99.65999 | ± | 0.02852 | 3 | 99.67844 | ± | 0.00599 | 3 | 99.65994 | ± | 0.01118 |
| 43 | 2 | 4 | 1 | Mg | Schist | shoot |  |  |  |  |  |  |  |  |  |  |  |  | 3 | 0.00073 | ± | 0.00013 | 3 | 0.00064 | ± | 0.00013 |
| 44 | 2 | 4 | 2 | Mg | Schist | root |  |  |  |  |  |  |  |  |  |  |  |  | 3 | 0.00080 | ± | 0.00007 | 3 | 0.00078 | ± | 0.00003 |
| 45 | 2 | 4 | 3 | Mg | Schist | water |  |  |  |  | 3 | 0.00823 | ± | 0.00063 | 3 | 0.00433 | ± | 0.00030 | 3 | 0.00964 | ± | 0.00113 | 3 | 0.00619 | ± | 0.00072 |
| 46 | 2 | 4 | 4 | Mg | Schist | aa | 3 | 0.08788 | ± | 0.00480 | 3 | 0.03608 | ± | 0.00096 | 3 | 0.03607 | ± | 0.00215 | 3 | 0.05475 | ± | 0.00262 | 3 | 0.04483 | ± | 0.00375 |
| 47 | 2 | 4 | 5 | Mg | Schist | ao | 3 | 0.08629 | ± | 0.00360 | 3 | 0.01060 | ± | 0.00049 | 3 | 0.06608 | ± | 0.05469 | 3 | 0.03597 | ± | 0.00166 | 3 | 0.02516 | ± | 0.00703 |
| 48 | 2 | 4 | 6 | Mg | Schist | unextracted | 3 | 99.82582 | ± | 0.00427 | 3 | 99.94508 | ± | 0.00134 | 3 | 99.89352 | ± | 0.05687 | 3 | 99.89812 | ± | 0.00138 | 3 | 99.92240 | ± | 0.01161 |
| 49 | 3 | 1 | 1 | Al | Basalt | shoot |  |  |  |  |  |  |  |  |  |  |  |  | 3 | 0.00002 | ± | 0.00000 | 3 | 0.00007 | ± | 0.00004 |
| 50 | 3 | 1 | 2 | Al | Basalt | root |  |  |  |  |  |  |  |  |  |  |  |  | 3 | 0.00012 | ± | 0.00003 | 3 | 0.00018 | ± | 0.00002 |
| 51 | 3 | 1 | 3 | Al | Basalt | water |  |  |  |  | 3 | 0.00007 | ± | 0.00000 | 3 | 0.00005 | ± | 0.00001 | 3 | 0.00005 | ± | 0.00000 | 3 | 0.00004 | ± | 0.00001 |
| 52 | 3 | 1 | 4 | Al | Basalt | aa | 3 | 0.04061 | ± | 0.00048 | 3 | 0.04537 | ± | 0.00067 | 3 | 0.04631 | ± | 0.00070 | 3 | 0.04746 | ± | 0.00086 | 3 | 0.04794 | ± | 0.00061 |
| 53 | 3 | 1 | 5 | Al | Basalt | ao | 3 | 2.67454 | ± | 0.01729 | 3 | 1.56041 | ± | 0.06087 | 3 | 1.58567 | ± | 0.19502 | 3 | 1.54218 | ± | 0.02793 | 3 | 1.74974 | ± | 0.19157 |
| 54 | 3 | 1 | 6 | Al | Basalt | unextracted | 3 | 97.28484 | ± | 0.01730 | 3 | 98.39415 | ± | 0.06119 | 3 | 98.36796 | ± | 0.19527 | 3 | 98.41016 | ± | 0.02871 | 3 | 98.20203 | ± | 0.19196 |
| 55 | 3 | 2 | 1 | Al | Rhyolite | shoot |  |  |  |  |  |  |  |  |  |  |  |  | 3 | 0.00001 | ± | 0.00001 | 3 | 0.00001 | ± | 0.00000 |
| 56 | 3 | 2 | 2 | Al | Rhyolite | root |  |  |  |  |  |  |  |  |  |  |  |  | 3 | 0.00003 | ± | 0.00001 | 3 | 0.00005 | ± | 0.00001 |
| 57 | 3 | 2 | 3 | Al | Rhyolite | water |  |  |  |  | 3 | 0.00001 | ± | 0.00000 | 3 | 0.00001 | ± | 0.00000 | 3 | 0.00003 | ± | 0.00001 | 3 | 0.00001 | ± | 0.00000 |
| 58 | 3 | 2 | 4 | Al | Rhyolite | aa | 3 | 0.01926 | ± | 0.00038 | 3 | 0.02264 | ± | 0.00093 | 3 | 0.03327 | ± | 0.00136 | 3 | 0.03030 | ± | 0.00204 | 3 | 0.02320 | ± | 0.00036 |
| 59 | 3 | 2 | 5 | Al | Rhyolite | ao | 3 | 0.16632 | ± | 0.00440 | 3 | 0.16221 | ± | 0.00183 | 3 | 0.22756 | ± | 0.00378 | 3 | 0.22135 | ± | 0.01934 | 3 | 0.16690 | ± | 0.00148 |
| 60 | 3 | 2 | 6 | Al | Rhyolite | unextracted | 3 | 99.81442 | ± | 0.00476 | 3 | 99.81514 | ± | 0.00267 | 3 | 99.73916 | ± | 0.00502 | 3 | 99.74828 | ± | 0.02093 | 3 | 99.80983 | ± | 0.00183 |
| 61 | 3 | 3 | 1 | Al | Granite | shoot |  |  |  |  |  |  |  |  |  |  |  |  | 3 | 0.00003 | ± | 0.00000 | 3 | 0.00002 | ± | 0.00001 |
| 62 | 3 | 3 | 2 | Al | Granite | root |  |  |  |  |  |  |  |  |  |  |  |  | 3 | 0.00003 | ± | 0.00001 | 3 | 0.00005 | ± | 0.00000 |
| 63 | 3 | 3 | 3 | Al | Granite | water |  |  |  |  | 3 | 0.00002 | ± | 0.00000 | 3 | 0.00002 | ± | 0.00000 | 3 | 0.00002 | ± | 0.00000 | 3 | 0.00002 | ± | 0.00000 |
| 64 | 3 | 3 | 4 | Al | Granite | aa | 3 | 0.01086 | ± | 0.00028 | 3 | 0.00506 | ± | 0.00008 | 3 | 0.00762 | ± | 0.00275 | 3 | 0.00531 | ± | 0.00007 | 3 | 0.00640 | ± | 0.00016 |
| 65 | 3 | 3 | 5 | Al | Granite | ao | 3 | 0.10134 | ± | 0.00220 | 3 | 0.04227 | ± | 0.00169 | 3 | 0.03872 | ± | 0.00188 | 3 | 0.04468 | ± | 0.00130 | 3 | 0.04607 | ± | 0.00242 |
| 66 | 3 | 3 | 6 | Al | Granite | unextracted | 3 | 99.88780 | ± | 0.00242 | 3 | 99.95265 | ± | 0.00172 | 3 | 99.95364 | ± | 0.00105 | 3 | 99.94994 | ± | 0.00123 | 3 | 99.94745 | ± | 0.00257 |
| 67 | 3 | 4 | 1 | Al | Schist | shoot |  |  |  |  |  |  |  |  |  |  |  |  | 3 | 0.00002 | ± | 0.00000 | 3 | 0.00002 | ± | 0.00001 |
| 68 | 3 | 4 | 2 | Al | Schist | root |  |  |  |  |  |  |  |  |  |  |  |  | 3 | 0.00011 | ± | 0.00001 | 3 | 0.00010 | ± | 0.00002 |
| 69 | 3 | 4 | 3 | Al | Schist | water |  |  |  |  | 3 | 0.00007 | ± | 0.00001 | 3 | 0.00004 | ± | 0.00000 | 3 | 0.00004 | ± | 0.00000 | 3 | 0.00005 | ± | 0.00000 |
| 70 | 3 | 4 | 4 | Al | Schist | aa | 3 | 0.01464 | ± | 0.00027 | 3 | 0.00369 | ± | 0.00048 | 3 | 0.00366 | ± | 0.00057 | 3 | 0.00691 | ± | 0.00022 | 3 | 0.00487 | ± | 0.00102 |
| 71 | 3 | 4 | 5 | Al | Schist | ao | 3 | 0.05839 | ± | 0.00154 | 3 | 0.01129 | ± | 0.00014 | 3 | 0.01262 | ± | 0.00065 | 3 | 0.04006 | ± | 0.00128 | 3 | 0.02597 | ± | 0.00763 |
| 72 | 3 | 4 | 6 | Al | Schist | unextracted | 3 | 99.92697 | ± | 0.00166 | 3 | 99.98494 | ± | 0.00036 | 3 | 99.98368 | ± | 0.00118 | 3 | 99.95286 | ± | 0.00145 | 3 | 99.96899 | ± | 0.00866 |
| 73 | 4 | 1 | 1 | Si | Basalt | shoot |  |  |  |  |  |  |  |  |  |  |  |  | 3 | 0.00008 | ± | 0.00002 | 3 | 0.00041 | ± | 0.00012 |
| 74 | 4 | 1 | 2 | Si | Basalt | root |  |  |  |  |  |  |  |  |  |  |  |  | 3 | 0.00010 | ± | 0.00002 | 3 | 0.00013 | ± | 0.00002 |
| 75 | 4 | 1 | 3 | Si | Basalt | water |  |  |  |  | 3 | 0.00461 | ± | 0.00009 | 3 | 0.00411 | ± | 0.00018 | 3 | 0.00387 | ± | 0.00030 | 3 | 0.00415 | ± | 0.00049 |
| 76 | 4 | 1 | 4 | Si | Basalt | aa | 3 | 0.03015 | ± | 0.00020 | 3 | 0.01092 | ± | 0.00048 | 3 | 0.01059 | ± | 0.00055 | 3 | 0.01180 | ± | 0.00062 | 3 | 0.01176 | ± | 0.00065 |
| 77 | 4 | 1 | 5 | Si | Basalt | ao | 3 | 2.32300 | ± | 0.04205 | 3 | 1.10731 | ± | 0.04107 | 3 | 1.10846 | ± | 0.15526 | 3 | 1.09096 | ± | 0.02890 | 3 | 1.24052 | ± | 0.13108 |
| 78 | 4 | 1 | 6 | Si | Basalt | unextracted | 3 | 97.64685 | ± | 0.04225 | 3 | 98.87716 | ± | 0.04141 | 3 | 98.87684 | ± | 0.15565 | 3 | 98.89318 | ± | 0.02905 | 3 | 98.74302 | ± | 0.13114 |
| 79 | 4 | 2 | 1 | Si | Rhyolite | shoot |  |  |  |  |  |  |  |  |  |  |  |  | 3 | 0.00004 | ± | 0.00001 | 3 | 0.00005 | ± | 0.00001 |
| 80 | 4 | 2 | 2 | Si | Rhyolite | root |  |  |  |  |  |  |  |  |  |  |  |  | 3 | 0.00005 | ± | 0.00002 | 3 | 0.00007 | ± | 0.00002 |
| 81 | 4 | 2 | 3 | Si | Rhyolite | water |  |  |  |  | 3 | 0.00410 | ± | 0.00019 | 3 | 0.00528 | ± | 0.00016 | 3 | 0.00567 | ± | 0.00020 | 3 | 0.00379 | ± | 0.00012 |
| 82 | 4 | 2 | 4 | Si | Rhyolite | aa | 3 | 0.01463 | ± | 0.00026 | 3 | 0.00412 | ± | 0.00033 | 3 | 0.00515 | ± | 0.00021 | 3 | 0.00441 | ± | 0.00022 | 3 | 0.00292 | ± | 0.00035 |
| 83 | 4 | 2 | 5 | Si | Rhyolite | ao | 3 | 0.02190 | ± | 0.00042 | 3 | 0.02663 | ± | 0.00053 | 3 | 0.02792 | ± | 0.00022 | 3 | 0.02842 | ± | 0.00072 | 3 | 0.02625 | ± | 0.00078 |
| 84 | 4 | 2 | 6 | Si | Rhyolite | unextracted | 3 | 99.96347 | ± | 0.00068 | 3 | 99.96515 | ± | 0.00046 | 3 | 99.96165 | ± | 0.00029 | 3 | 99.96141 | ± | 0.00070 | 3 | 99.96692 | ± | 0.00096 |
| 85 | 4 | 3 | 1 | Si | Granite | shoot |  |  |  |  |  |  |  |  |  |  |  |  | 3 | 0.00003 | ± | 0.00001 | 3 | 0.00007 | ± | 0.00001 |
| 86 | 4 | 3 | 2 | Si | Granite | root |  |  |  |  |  |  |  |  |  |  |  |  | 3 | 0.00007 | ± | 0.00003 | 3 | 0.00004 | ± | 0.00000 |
| 87 | 4 | 3 | 3 | Si | Granite | water |  |  |  |  | 3 | 0.00237 | ± | 0.00005 | 3 | 0.00229 | ± | 0.00006 | 3 | 0.00201 | ± | 0.00008 | 3 | 0.00232 | ± | 0.00005 |
| 88 | 4 | 3 | 4 | Si | Granite | aa | 3 | 0.01210 | ± | 0.00019 | 3 | 0.00034 | ± | 0.00005 | 3 | 0.00041 | ± | 0.00018 | 1 | 0.00054 |  |  | 3 | 0.00087 | ± | 0.00005 |
| 89 | 4 | 3 | 5 | Si | Granite | ao | 3 | 0.02350 | ± | 0.00032 | 3 | 0.01479 | ± | 0.00112 | 3 | 0.01474 | ± | 0.00111 | 3 | 0.01574 | ± | 0.00006 | 3 | 0.01700 | ± | 0.00076 |
| 90 | 4 | 3 | 6 | Si | Granite | unextracted | 3 | 99.96440 | ± | 0.00041 | 3 | 99.98250 | ± | 0.00112 | 3 | 99.98256 | ± | 0.00097 | 3 | 99.98197 | ± | 0.00010 | 3 | 99.97970 | ± | 0.00081 |
| 91 | 4 | 4 | 1 | Si | Schist | shoot |  |  |  |  |  |  |  |  |  |  |  |  | 3 | 0.00009 | ± | 0.00001 | 3 | 0.00009 | ± | 0.00001 |
| 92 | 4 | 4 | 2 | Si | Schist | root |  |  |  |  |  |  |  |  |  |  |  |  | 3 | 0.00005 | ± | 0.00001 | 3 | 0.00006 | ± | 0.00001 |
| 93 | 4 | 4 | 3 | Si | Schist | water |  |  |  |  | 3 | 0.00182 | ± | 0.00011 | 3 | 0.00158 | ± | 0.00002 | 3 | 0.00369 | ± | 0.00020 | 3 | 0.00240 | ± | 0.00042 |
| 94 | 4 | 4 | 4 | Si | Schist | aa | 3 | 0.01447 | ± | 0.00042 | 3 | 0.00183 | ± | 0.00020 | 3 | 0.00129 | ± | 0.00017 | 3 | 0.00116 | ± | 0.00011 | 3 | 0.00036 | ± | 0.00018 |
| 95 | 4 | 4 | 5 | Si | Schist | ao | 3 | 0.01495 | ± | 0.00035 | 3 | 0.00853 | ± | 0.00051 | 3 | 0.01189 | ± | 0.00289 | 3 | 0.01231 | ± | 0.00024 | 3 | 0.01042 | ± | 0.00106 |
| 96 | 4 | 4 | 6 | Si | Schist | unextracted | 3 | 99.97058 | ± | 0.00066 | 3 | 99.98782 | ± | 0.00036 | 3 | 99.98524 | ± | 0.00275 | 3 | 99.98270 | ± | 0.00040 | 3 | 99.98667 | ± | 0.00136 |
| 97 | 5 | 1 | 1 | P | Basalt | shoot |  |  |  |  |  |  |  |  |  |  |  |  | 3 | 0.00213 | ± | 0.00054 | 3 | 0.00273 | ± | 0.00031 |
| 98 | 5 | 1 | 2 | P | Basalt | root |  |  |  |  |  |  |  |  |  |  |  |  | 3 | 0.00317 | ± | 0.00062 | 3 | 0.00456 | ± | 0.00073 |
| 99 | 5 | 1 | 3 | P | Basalt | water |  |  |  |  | 3 | 0.00664 | ± | 0.00058 | 3 | 0.00651 | ± | 0.00065 | 3 | 0.00579 | ± | 0.00016 | 3 | 0.00503 | ± | 0.00020 |
| 100 | 5 | 1 | 4 | P | Basalt | aa | 3 | 0.72124 | ± | 0.00700 | 3 | 0.47358 | ± | 0.00841 | 3 | 0.45043 | ± | 0.00535 | 3 | 0.46298 | ± | 0.01700 | 3 | 0.46713 | ± | 0.01753 |
| 101 | 5 | 1 | 5 | P | Basalt | ao | 3 | 9.55321 | ± | 0.19719 | 3 | 5.33289 | ± | 0.25761 | 3 | 6.08242 | ± | 0.13371 | 3 | 5.80246 | ± | 0.07339 | 3 | 6.39989 | ± | 0.38527 |
| 102 | 5 | 1 | 6 | P | Basalt | unextracted | 3 | 89.72555 | ± | 0.20249 | 3 | 94.18690 | ± | 0.26379 | 3 | 93.46064 | ± | 0.12902 | 3 | 93.72349 | ± | 0.08904 | 3 | 93.12067 | ± | 0.40114 |
| 103 | 5 | 2 | 1 | P | Rhyolite | shoot |  |  |  |  |  |  |  |  |  |  |  |  | 3 | 0.02335 | ± | 0.00667 | 3 | 0.03896 | ± | 0.01457 |
| 104 | 5 | 2 | 2 | P | Rhyolite | root |  |  |  |  |  |  |  |  |  |  |  |  | 3 | 0.02664 | ± | 0.00645 | 3 | 0.03355 | ± | 0.00697 |
| 105 | 5 | 2 | 3 | P | Rhyolite | water |  |  |  |  | 3 | 0.03347 | ± | 0.00368 | 3 | 0.08136 | ± | 0.00612 | 3 | 0.07399 | ± | 0.01308 | 3 | 0.02978 | ± | 0.00340 |
| 106 | 5 | 2 | 4 | P | Rhyolite | aa | 3 | 1.58164 | ± | 0.16217 | 3 | 1.18135 | ± | 0.08198 | 3 | 0.42494 | ± | 0.03161 | 3 | 0.71397 | ± | 0.05275 | 3 | 0.72043 | ± | 0.16297 |
| 107 | 5 | 2 | 5 | P | Rhyolite | ao | 3 | 17.48938 | ± | 0.53915 | 3 | 13.73536 | ± | 0.58663 | 3 | 15.91596 | ± | 0.52030 | 3 | 15.19889 | ± | 0.12052 | 3 | 14.26216 | ± | 0.89916 |
| 108 | 5 | 2 | 6 | P | Rhyolite | unextracted | 3 | 80.92898 | ± | 0.38550 | 3 | 85.04981 | ± | 0.66301 | 3 | 83.57775 | ± | 0.50358 | 3 | 83.96317 | ± | 0.12736 | 3 | 84.91512 | ± | 1.00831 |
| 109 | 5 | 3 | 1 | P | Granite | shoot |  |  |  |  |  |  |  |  |  |  |  |  | 3 | 0.01471 | ± | 0.00294 | 3 | 0.01471 | ± | 0.00046 |
| 110 | 5 | 3 | 2 | P | Granite | root |  |  |  |  |  |  |  |  |  |  |  |  | 3 | 0.02066 | ± | 0.00591 | 3 | 0.02273 | ± | 0.00266 |
| 111 | 5 | 3 | 3 | P | Granite | water |  |  |  |  | 3 | 0.01523 | ± | 0.00307 | 3 | 0.01733 | ± | 0.00298 | 3 | 0.01725 | ± | 0.00194 | 3 | 0.02485 | ± | 0.00383 |
| 112 | 5 | 3 | 4 | P | Granite | aa | 3 | 1.49371 | ± | 0.09345 | 3 | 0.39758 | ± | 0.07281 | 3 | 0.31223 | ± | 0.07732 | 3 | 0.27763 | ± | 0.01170 | 3 | 0.41283 | ± | 0.02592 |
| 113 | 5 | 3 | 5 | P | Granite | ao | 3 | 16.94637 | ± | 0.98793 | 3 | 11.98125 | ± | 0.19107 | 3 | 11.32337 | ± | 0.67795 | 3 | 11.84283 | ± | 0.37940 | 3 | 10.26303 | ± | 0.53384 |
| 114 | 5 | 3 | 6 | P | Granite | unextracted | 3 | 81.55991 | ± | 0.96629 | 3 | 87.60595 | ± | 0.13918 | 3 | 88.34707 | ± | 0.62986 | 3 | 87.82691 | ± | 0.38083 | 3 | 89.26185 | ± | 0.51160 |
| 115 | 5 | 4 | 1 | P | Schist | shoot |  |  |  |  |  |  |  |  |  |  |  |  | 3 | 0.05654 | ± | 0.00904 | 3 | 0.05293 | ± | 0.00556 |
| 116 | 5 | 4 | 2 | P | Schist | root |  |  |  |  |  |  |  |  |  |  |  |  | 3 | 0.05896 | ± | 0.00932 | 3 | 0.05845 | ± | 0.00311 |
| 117 | 5 | 4 | 3 | P | Schist | water |  |  |  |  | 3 | 0.08861 | ± | 0.01416 | 3 | 0.09396 | ± | 0.00253 | 3 | 0.05060 | ± | 0.00524 | 3 | 0.07977 | ± | 0.01885 |
| 118 | 5 | 4 | 4 | P | Schist | aa | 3 | 2.10413 | ± | 0.04803 | 3 | 0.18292 | ± | 0.09990 | 3 | 0.34955 | ± | 0.05436 | 3 | 0.82793 | ± | 0.04172 | 3 | 0.26291 | ± | 0.10255 |
| 119 | 5 | 4 | 5 | P | Schist | ao | 3 | 12.17993 | ± | 0.89321 | 3 | 18.53481 | ± | 0.65225 | 3 | 20.33146 | ± | 0.60153 | 3 | 21.07635 | ± | 1.52255 | 3 | 22.48031 | ± | 0.82520 |
| 120 | 5 | 4 | 6 | P | Schist | unextracted | 3 | 85.71594 | ± | 0.89486 | 3 | 81.19365 | ± | 0.71867 | 3 | 79.22502 | ± | 0.56107 | 3 | 77.92963 | ± | 1.50107 | 3 | 77.06563 | ± | 0.74246 |
| 121 | 6 | 1 | 1 | K | Basalt | shoot |  |  |  |  |  |  |  |  |  |  |  |  | 3 | 0.00020 | ± | 0.00013 | 3 | 0.00036 | ± | 0.00017 |
| 122 | 6 | 1 | 2 | K | Basalt | root |  |  |  |  |  |  |  |  |  |  |  |  | 3 | 0.00201 | ± | 0.00187 | 3 | 0.00304 | ± | 0.00205 |
| 123 | 6 | 1 | 3 | K | Basalt | water |  |  |  |  | 3 | 0.03243 | ± | 0.00027 | 3 | 0.03038 | ± | 0.00019 | 3 | 0.03208 | ± | 0.00124 | 3 | 0.03405 | ± | 0.00282 |
| 124 | 6 | 1 | 4 | K | Basalt | aa | 3 | 0.32224 | ± | 0.00367 | 3 | 0.31966 | ± | 0.01202 | 3 | 0.29020 | ± | 0.00289 | 3 | 0.29870 | ± | 0.01177 | 3 | 0.28833 | ± | 0.00756 |
| 125 | 6 | 1 | 5 | K | Basalt | ao | 3 | 9.76332 | ± | 0.00462 | 3 | 6.09482 | ± | 0.31491 | 3 | 6.47188 | ± | 0.12948 | 3 | 6.43077 | ± | 0.16314 | 3 | 6.90077 | ± | 0.36759 |
| 126 | 6 | 1 | 6 | K | Basalt | unextracted | 3 | 89.91445 | ± | 0.00095 | 3 | 93.55309 | ± | 0.32482 | 3 | 93.20753 | ± | 0.12788 | 3 | 93.23624 | ± | 0.15957 | 3 | 92.77345 | ± | 0.37526 |
| 127 | 6 | 2 | 1 | K | Rhyolite | shoot |  |  |  |  |  |  |  |  |  |  |  |  | 3 | 0.00052 | ± | 0.00010 | 3 | 0.00061 | ± | 0.00015 |
| 128 | 6 | 2 | 2 | K | Rhyolite | root |  |  |  |  |  |  |  |  |  |  |  |  | 3 | 0.00080 | ± | 0.00023 | 3 | 0.00114 | ± | 0.00034 |
| 129 | 6 | 2 | 3 | K | Rhyolite | water |  |  |  |  | 3 | 0.00473 | ± | 0.00029 | 3 | 0.00642 | ± | 0.00028 | 3 | 0.00716 | ± | 0.00023 | 3 | 0.00595 | ± | 0.00024 |
| 130 | 6 | 2 | 4 | K | Rhyolite | aa | 3 | 0.08869 | ± | 0.00149 | 3 | 0.08515 | ± | 0.00465 | 3 | 0.12736 | ± | 0.00456 | 3 | 0.12447 | ± | 0.01199 | 3 | 0.08417 | ± | 0.00075 |
| 131 | 6 | 2 | 5 | K | Rhyolite | ao | 3 | 0.57683 | ± | 0.01108 | 3 | 0.54212 | ± | 0.01141 | 3 | 0.49889 | ± | 0.00516 | 3 | 0.51999 | ± | 0.00110 | 3 | 0.51265 | ± | 0.00207 |
| 132 | 6 | 2 | 6 | K | Rhyolite | unextracted | 3 | 99.33448 | ± | 0.01256 | 3 | 99.36800 | ± | 0.01441 | 3 | 99.36733 | ± | 0.00091 | 3 | 99.34707 | ± | 0.01181 | 3 | 99.39548 | ± | 0.00322 |
| 133 | 6 | 3 | 1 | K | Granite | shoot |  |  |  |  |  |  |  |  |  |  |  |  | 3 | 0.00046 | ± | 0.00002 | 3 | 0.00059 | ± | 0.00003 |
| 134 | 6 | 3 | 2 | K | Granite | root |  |  |  |  |  |  |  |  |  |  |  |  | 3 | 0.00072 | ± | 0.00026 | 3 | 0.00075 | ± | 0.00014 |
| 135 | 6 | 3 | 3 | K | Granite | water |  |  |  |  | 3 | 0.01243 | ± | 0.00039 | 3 | 0.01092 | ± | 0.00012 | 3 | 0.01433 | ± | 0.00118 | 3 | 0.01545 | ± | 0.00088 |
| 136 | 6 | 3 | 4 | K | Granite | aa | 3 | 0.04199 | ± | 0.00116 | 3 | 0.01984 | ± | 0.00093 | 3 | 0.01971 | ± | 0.00111 | 3 | 0.01995 | ± | 0.00078 | 3 | 0.02458 | ± | 0.00167 |
| 137 | 6 | 3 | 5 | K | Granite | ao | 3 | 0.59933 | ± | 0.00531 | 3 | 0.57797 | ± | 0.00589 | 3 | 0.57481 | ± | 0.01405 | 3 | 0.57082 | ± | 0.00684 | 3 | 0.58912 | ± | 0.01037 |
| 138 | 6 | 3 | 6 | K | Granite | unextracted | 3 | 99.35868 | ± | 0.00644 | 3 | 99.38977 | ± | 0.00660 | 3 | 99.39456 | ± | 0.01338 | 3 | 99.39372 | ± | 0.00702 | 3 | 99.36951 | ± | 0.01211 |
| 139 | 6 | 4 | 1 | K | Schist | shoot |  |  |  |  |  |  |  |  |  |  |  |  | 3 | 0.00053 | ± | 0.00016 | 3 | 0.00056 | ± | 0.00015 |
| 140 | 6 | 4 | 2 | K | Schist | root |  |  |  |  |  |  |  |  |  |  |  |  | 3 | 0.00064 | ± | 0.00017 | 3 | 0.00052 | ± | 0.00011 |
| 141 | 6 | 4 | 3 | K | Schist | water |  |  |  |  | 3 | 0.00653 | ± | 0.00024 | 3 | 0.00462 | ± | 0.00016 | 3 | 0.01227 | ± | 0.00067 | 3 | 0.00852 | ± | 0.00123 |
| 142 | 6 | 4 | 4 | K | Schist | aa | 3 | 0.03562 | ± | 0.00103 | 3 | 0.01083 | ± | 0.00082 | 3 | 0.01133 | ± | 0.00105 | 3 | 0.02944 | ± | 0.00053 | 3 | 0.01927 | ± | 0.00468 |
| 143 | 6 | 4 | 5 | K | Schist | ao | 3 | 0.39492 | ± | 0.00753 | 3 | 0.36982 | ± | 0.00464 | 3 | 0.37464 | ± | 0.00340 | 3 | 0.38004 | ± | 0.00319 | 3 | 0.36469 | ± | 0.00474 |
| 144 | 6 | 4 | 6 | K | Schist | unextracted | 3 | 99.56946 | ± | 0.00850 | 3 | 99.61281 | ± | 0.00530 | 3 | 99.60941 | ± | 0.00226 | 3 | 99.57708 | ± | 0.00360 | 3 | 99.60645 | ± | 0.01041 |
| 145 | 7 | 1 | 1 | Ca | Basalt | shoot |  |  |  |  |  |  |  |  |  |  |  |  | 3 | 0.00106 | ± | 0.00026 | 3 | 0.00122 | ± | 0.00025 |
| 146 | 7 | 1 | 2 | Ca | Basalt | root |  |  |  |  |  |  |  |  |  |  |  |  | 3 | 0.00139 | ± | 0.00016 | 3 | 0.00164 | ± | 0.00014 |
| 147 | 7 | 1 | 3 | Ca | Basalt | water |  |  |  |  | 3 | 0.02965 | ± | 0.00118 | 3 | 0.03446 | ± | 0.00195 | 3 | 0.04121 | ± | 0.00271 | 3 | 0.04891 | ± | 0.00638 |
| 148 | 7 | 1 | 4 | Ca | Basalt | aa | 3 | 0.90106 | ± | 0.05545 | 3 | 0.43142 | ± | 0.03720 | 3 | 0.44636 | ± | 0.03804 | 3 | 0.45467 | ± | 0.07379 | 3 | 0.42138 | ± | 0.04294 |
| 149 | 7 | 1 | 5 | Ca | Basalt | ao | 3 | 0.06656 | ± | 0.00613 | 3 | 0.08842 | ± | 0.00654 | 3 | 0.09328 | ± | 0.00176 | 3 | 0.10087 | ± | 0.00847 | 3 | 0.09450 | ± | 0.00249 |
| 150 | 7 | 1 | 6 | Ca | Basalt | unextracted | 3 | 99.03238 | ± | 0.06080 | 3 | 99.45052 | ± | 0.04300 | 3 | 99.42590 | ± | 0.03678 | 3 | 99.40080 | ± | 0.07965 | 3 | 99.43235 | ± | 0.04453 |
| 151 | 7 | 2 | 1 | Ca | Rhyolite | shoot |  |  |  |  |  |  |  |  |  |  |  |  | 3 | 0.00775 | ± | 0.00158 | 3 | 0.01481 | ± | 0.00358 |
| 152 | 7 | 2 | 2 | Ca | Rhyolite | root |  |  |  |  |  |  |  |  |  |  |  |  | 3 | 0.00945 | ± | 0.00139 | 3 | 0.01391 | ± | 0.00302 |
| 153 | 7 | 2 | 3 | Ca | Rhyolite | water |  |  |  |  | 3 | 0.24353 | ± | 0.03068 | 3 | 0.20271 | ± | 0.01991 | 3 | 0.21935 | ± | 0.04183 | 3 | 0.27708 | ± | 0.01080 |
| 154 | 7 | 2 | 4 | Ca | Rhyolite | aa | 3 | 11.84950 | ± | 0.29141 | 3 | 7.98953 | ± | 0.14047 | 3 | 8.23971 | ± | 0.29495 | 3 | 7.52836 | ± | 0.27066 | 3 | 6.66611 | ± | 0.21646 |
| 155 | 7 | 2 | 5 | Ca | Rhyolite | ao | 3 | 0.73299 | ± | 0.05768 | 3 | 1.39833 | ± | 0.65383 | 3 | 0.61787 | ± | 0.00648 | 3 | 0.69582 | ± | 0.01796 | 3 | 0.73227 | ± | 0.08620 |
| 156 | 7 | 2 | 6 | Ca | Rhyolite | unextracted | 3 | 87.41752 | ± | 0.31016 | 3 | 90.36862 | ± | 0.79803 | 3 | 90.93970 | ± | 0.28078 | 3 | 91.53927 | ± | 0.22747 | 3 | 92.29582 | ± | 0.29402 |
| 157 | 7 | 3 | 1 | Ca | Granite | shoot |  |  |  |  |  |  |  |  |  |  |  |  | 3 | 0.00318 | ± | 0.00052 | 3 | 0.00437 | ± | 0.00045 |
| 158 | 7 | 3 | 2 | Ca | Granite | root |  |  |  |  |  |  |  |  |  |  |  |  | 3 | 0.00608 | ± | 0.00219 | 3 | 0.00362 | ± | 0.00055 |
| 159 | 7 | 3 | 3 | Ca | Granite | water |  |  |  |  | 3 | 0.31430 | ± | 0.00880 | 3 | 0.24020 | ± | 0.01025 | 3 | 0.38381 | ± | 0.03786 | 3 | 0.41311 | ± | 0.05115 |
| 160 | 7 | 3 | 4 | Ca | Granite | aa | 3 | 6.14749 | ± | 0.22641 | 3 | 2.70519 | ± | 0.32088 | 3 | 2.36068 | ± | 0.06770 | 3 | 2.09349 | ± | 0.10424 | 3 | 2.42491 | ± | 0.27476 |
| 161 | 7 | 3 | 5 | Ca | Granite | ao | 3 | 0.07811 | ± | 0.01566 | 3 | 0.23199 | ± | 0.01610 | 3 | 0.19313 | ± | 0.00503 | 3 | 0.21078 | ± | 0.00736 | 3 | 0.29555 | ± | 0.00544 |
| 162 | 7 | 3 | 6 | Ca | Granite | unextracted | 3 | 93.77440 | ± | 0.23861 | 3 | 96.74852 | ± | 0.32763 | 3 | 97.20599 | ± | 0.08044 | 3 | 97.30266 | ± | 0.08601 | 3 | 96.85845 | ± | 0.23597 |
| 163 | 7 | 4 | 1 | Ca | Schist | shoot |  |  |  |  |  |  |  |  |  |  |  |  | 3 | 0.08203 | ± | 0.01681 | 3 | 0.07139 | ± | 0.01304 |
| 164 | 7 | 4 | 2 | Ca | Schist | root |  |  |  |  |  |  |  |  |  |  |  |  | 3 | 0.07403 | ± | 0.01543 | 3 | 0.06613 | ± | 0.01219 |
| 165 | 7 | 4 | 3 | Ca | Schist | water |  |  |  |  | 3 | 1.05398 | ± | 0.05584 | 3 | 0.63133 | ± | 0.02580 | 3 | 1.10283 | ± | 0.09604 | 3 | 0.84125 | ± | 0.04151 |
| 166 | 7 | 4 | 4 | Ca | Schist | aa | 3 | 24.98316 | ± | 5.40112 | 3 | 6.68149 | ± | 0.13003 | 3 | 6.59238 | ± | 0.07817 | 3 | 8.76629 | ± | 0.35816 | 3 | 9.80685 | ± | 1.71631 |
| 167 | 7 | 4 | 5 | Ca | Schist | ao | 3 | 2.73797 | ± | 1.47272 | 3 | 3.40444 | ± | 0.27740 | 3 | 3.60663 | ± | 0.10499 | 3 | 3.84416 | ± | 0.09955 | 3 | 3.39673 | ± | 0.02608 |
| 168 | 7 | 4 | 6 | Ca | Schist | unextracted | 3 | 72.27887 | ± | 4.94515 | 3 | 88.86009 | ± | 0.38736 | 3 | 89.16966 | ± | 0.11845 | 3 | 86.13066 | ± | 0.39176 | 3 | 85.81765 | ± | 1.80142 |
| 169 | 8 | 1 | 1 | Ti | Basalt | shoot |  |  |  |  |  |  |  |  |  |  |  |  | 3 | 0.00001 | ± | 0.00000 | 3 | 0.00009 | ± | 0.00006 |
| 170 | 8 | 1 | 2 | Ti | Basalt | root |  |  |  |  |  |  |  |  |  |  |  |  | 3 | 0.00020 | ± | 0.00003 | 3 | 0.00027 | ± | 0.00004 |
| 171 | 8 | 1 | 3 | Ti | Basalt | water |  |  |  |  | 3 | 0.00001 | ± | 0.00000 | 3 | 0.00001 | ± | 0.00000 | 3 | 0.00001 | ± | 0.00000 | 3 | 0.00001 | ± | 0.00000 |
| 172 | 8 | 1 | 4 | Ti | Basalt | aa | 3 | 0.00429 | ± | 0.00006 | 3 | 0.00233 | ± | 0.00010 | 3 | 0.00287 | ± | 0.00011 | 3 | 0.00291 | ± | 0.00020 | 3 | 0.00292 | ± | 0.00025 |
| 173 | 8 | 1 | 5 | Ti | Basalt | ao | 3 | 6.89495 | ± | 0.11438 | 3 | 4.23606 | ± | 0.16703 | 3 | 4.70033 | ± | 0.16606 | 3 | 4.40690 | ± | 0.05330 | 3 | 4.83926 | ± | 0.30263 |
| 174 | 8 | 1 | 6 | Ti | Basalt | unextracted | 3 | 93.10076 | ± | 0.11437 | 3 | 95.76160 | ± | 0.16698 | 3 | 95.29680 | ± | 0.16600 | 3 | 95.58996 | ± | 0.05336 | 3 | 95.15747 | ± | 0.30242 |
| 175 | 8 | 2 | 1 | Ti | Rhyolite | shoot |  |  |  |  |  |  |  |  |  |  |  |  | 3 | 0.00003 | ± | 0.00002 | 3 | 0.00001 | ± | 0.00000 |
| 176 | 8 | 2 | 2 | Ti | Rhyolite | root |  |  |  |  |  |  |  |  |  |  |  |  | 3 | 0.00008 | ± | 0.00002 | 3 | 0.00005 | ± | 0.00001 |
| 177 | 8 | 2 | 3 | Ti | Rhyolite | water |  |  |  |  | 3 | 0.00002 | ± | 0.00000 | 3 | 0.00003 | ± | 0.00000 | 3 | 0.00004 | ± | 0.00001 | 3 | 0.00001 | ± | 0.00000 |
| 178 | 8 | 2 | 4 | Ti | Rhyolite | aa | 3 | 0.00083 | ± | 0.00011 | 3 | 0.00107 | ± | 0.00065 | 3 | 0.00235 | ± | 0.00178 | 3 | 0.00024 | ± | 0.00014 | 3 | 0.00026 | ± | 0.00015 |
| 179 | 8 | 2 | 5 | Ti | Rhyolite | ao | 3 | 0.04274 | ± | 0.00105 | 3 | 0.05026 | ± | 0.00366 | 3 | 0.11457 | ± | 0.01319 | 3 | 0.09692 | ± | 0.01639 | 3 | 0.05434 | ± | 0.00035 |
| 180 | 8 | 2 | 6 | Ti | Rhyolite | unextracted | 3 | 99.95643 | ± | 0.00099 | 3 | 99.94866 | ± | 0.00311 | 3 | 99.88305 | ± | 0.01257 | 3 | 99.90269 | ± | 0.01655 | 3 | 99.94532 | ± | 0.00048 |
| 181 | 8 | 3 | 1 | Ti | Granite | shoot |  |  |  |  |  |  |  |  |  |  |  |  | 3 | 0.00012 | ± | 0.00002 | 3 | 0.00008 | ± | 0.00003 |
| 182 | 8 | 3 | 2 | Ti | Granite | root |  |  |  |  |  |  |  |  |  |  |  |  | 3 | 0.00016 | ± | 0.00005 | 3 | 0.00017 | ± | 0.00003 |
| 183 | 8 | 3 | 3 | Ti | Granite | water |  |  |  |  | 3 | 0.00001 | ± | 0.00000 | 3 | 0.00001 | ± | 0.00000 | 3 | 0.00001 | ± | 0.00000 | 3 | 0.00001 | ± | 0.00000 |
| 184 | 8 | 3 | 4 | Ti | Granite | aa | 3 | 0.00446 | ± | 0.00025 | 3 | 0.00150 | ± | 0.00013 | 3 | 0.00161 | ± | 0.00013 | 3 | 0.00128 | ± | 0.00002 | 3 | 0.00162 | ± | 0.00013 |
| 185 | 8 | 3 | 5 | Ti | Granite | ao | 3 | 0.21541 | ± | 0.01714 | 3 | 0.04786 | ± | 0.00722 | 3 | 0.04649 | ± | 0.00726 | 3 | 0.05701 | ± | 0.00452 | 3 | 0.05549 | ± | 0.00374 |
| 186 | 8 | 3 | 6 | Ti | Granite | unextracted | 3 | 99.78013 | ± | 0.01716 | 3 | 99.95062 | ± | 0.00717 | 3 | 99.95189 | ± | 0.00712 | 3 | 99.94142 | ± | 0.00454 | 3 | 99.94262 | ± | 0.00386 |
| 187 | 8 | 4 | 1 | Ti | Schist | shoot |  |  |  |  |  |  |  |  |  |  |  |  | 3 | 0.00003 | ± | 0.00001 | 3 | 0.00004 | ± | 0.00001 |
| 188 | 8 | 4 | 2 | Ti | Schist | root |  |  |  |  |  |  |  |  |  |  |  |  | 3 | 0.00022 | ± | 0.00002 | 3 | 0.00018 | ± | 0.00004 |
| 189 | 8 | 4 | 3 | Ti | Schist | water |  |  |  |  | 3 | 0.00006 | ± | 0.00000 | 3 | 0.00005 | ± | 0.00000 | 3 | 0.00007 | ± | 0.00001 | 3 | 0.00008 | ± | 0.00001 |
| 190 | 8 | 4 | 4 | Ti | Schist | aa | 3 | 0.00346 | ± | 0.00025 | 2 | 0.00039 | ± | 0.00016 | 3 | 0.00038 | ± | 0.00006 | 3 | 0.00116 | ± | 0.00011 | 3 | 0.00093 | ± | 0.00017 |
| 191 | 8 | 4 | 5 | Ti | Schist | ao | 3 | 0.07117 | ± | 0.00206 | 3 | 0.00868 | ± | 0.00151 | 3 | 0.01932 | ± | 0.01151 | 3 | 0.04384 | ± | 0.00097 | 3 | 0.02450 | ± | 0.00806 |
| 192 | 8 | 4 | 6 | Ti | Schist | unextracted | 3 | 99.92537 | ± | 0.00231 | 3 | 99.99100 | ± | 0.00152 | 3 | 99.98025 | ± | 0.01145 | 3 | 99.95469 | ± | 0.00093 | 3 | 99.97426 | ± | 0.00826 |
| 193 | 9 | 1 | 1 | Mn | Basalt | shoot |  |  |  |  |  |  |  |  |  |  |  |  | 3 | 0.00174 | ± | 0.00069 | 3 | 0.00306 | ± | 0.00068 |
| 194 | 9 | 1 | 2 | Mn | Basalt | root |  |  |  |  |  |  |  |  |  |  |  |  | 3 | 0.00148 | ± | 0.00030 | 3 | 0.00232 | ± | 0.00043 |
| 195 | 9 | 1 | 3 | Mn | Basalt | water |  |  |  |  | 3 | 0.00040 | ± | 0.00027 | 3 | 0.00081 | ± | 0.00026 | 3 | 0.00057 | ± | 0.00031 | 3 | 0.00222 | ± | 0.00115 |
| 196 | 9 | 1 | 4 | Mn | Basalt | aa | 3 | 0.13394 | ± | 0.00102 | 3 | 0.12103 | ± | 0.00163 | 3 | 0.13458 | ± | 0.00134 | 3 | 0.12768 | ± | 0.00078 | 3 | 0.13265 | ± | 0.01060 |
| 197 | 9 | 1 | 5 | Mn | Basalt | ao | 3 | 5.19660 | ± | 0.05481 | 3 | 2.94864 | ± | 0.13380 | 3 | 3.26902 | ± | 0.11890 | 3 | 3.09991 | ± | 0.01777 | 3 | 3.50626 | ± | 0.21153 |
| 198 | 9 | 1 | 6 | Mn | Basalt | unextracted | 3 | 94.66946 | ± | 0.05423 | 3 | 96.92992 | ± | 0.13471 | 3 | 96.59559 | ± | 0.11861 | 3 | 96.76862 | ± | 0.01978 | 3 | 96.35349 | ± | 0.21217 |
| 199 | 9 | 2 | 1 | Mn | Rhyolite | shoot |  |  |  |  |  |  |  |  |  |  |  |  | 3 | 0.04278 | ± | 0.00966 | 3 | 0.05212 | ± | 0.00666 |
| 200 | 9 | 2 | 2 | Mn | Rhyolite | root |  |  |  |  |  |  |  |  |  |  |  |  | 3 | 0.03312 | ± | 0.00601 | 3 | 0.03983 | ± | 0.00989 |
| 201 | 9 | 2 | 3 | Mn | Rhyolite | water |  |  |  |  | 3 | 0.07167 | ± | 0.05279 | 3 | 0.02557 | ± | 0.00483 | 3 | 0.04063 | ± | 0.01828 | 3 | 0.03651 | ± | 0.00292 |
| 202 | 9 | 2 | 4 | Mn | Rhyolite | aa | 3 | 3.26617 | ± | 0.42036 | 3 | 2.45942 | ± | 0.55968 | 3 | 1.72494 | ± | 0.09028 | 3 | 1.68700 | ± | 0.20006 | 3 | 1.80434 | ± | 0.17568 |
| 203 | 9 | 2 | 5 | Mn | Rhyolite | ao | 3 | 45.65611 | ± | 1.39764 | 3 | 37.77452 | ± | 1.60405 | 3 | 20.60621 | ± | 2.53077 | 3 | 19.06043 | ± | 2.95558 | 3 | 35.23366 | ± | 2.77762 |
| 204 | 9 | 2 | 6 | Mn | Rhyolite | unextracted | 3 | 51.07772 | ± | 1.04351 | 3 | 59.69439 | ± | 2.20426 | 3 | 77.64328 | ± | 2.61829 | 3 | 79.13604 | ± | 3.15928 | 3 | 62.83354 | ± | 2.67419 |
| 205 | 9 | 3 | 1 | Mn | Granite | shoot |  |  |  |  |  |  |  |  |  |  |  |  | 3 | 0.00527 | ± | 0.00097 | 3 | 0.00690 | ± | 0.00109 |
| 206 | 9 | 3 | 2 | Mn | Granite | root |  |  |  |  |  |  |  |  |  |  |  |  | 3 | 0.01037 | ± | 0.00325 | 3 | 0.00768 | ± | 0.00103 |
| 207 | 9 | 3 | 3 | Mn | Granite | water |  |  |  |  | 3 | 0.01169 | ± | 0.00123 | 3 | 0.00606 | ± | 0.00271 | 3 | 0.01059 | ± | 0.00418 | 3 | 0.01188 | ± | 0.00165 |
| 208 | 9 | 3 | 4 | Mn | Granite | aa | 3 | 1.02515 | ± | 0.04061 | 3 | 0.53762 | ± | 0.08178 | 3 | 0.55199 | ± | 0.08959 | 3 | 0.41244 | ± | 0.04945 | 3 | 0.52635 | ± | 0.09088 |
| 209 | 9 | 3 | 5 | Mn | Granite | ao | 3 | 1.29002 | ± | 0.08306 | 0 |  |  |  | 2 | 0.21622 | ± | 0.12600 | 1 | 0.09630 |  |  | 2 | 1.00938 | ± | 0.08345 |
| 210 | 9 | 3 | 6 | Mn | Granite | unextracted | 3 | 97.68483 | ± | 0.09942 | 3 | 99.45069 | ± | 0.08200 | 3 | 99.22573 | ± | 0.19068 | 3 | 99.46503 | ± | 0.06950 | 3 | 98.43781 | ± | 0.42806 |
| 211 | 9 | 4 | 1 | Mn | Schist | shoot |  |  |  |  |  |  |  |  |  |  |  |  | 3 | 0.01844 | ± | 0.00275 | 3 | 0.01973 | ± | 0.00315 |
| 212 | 9 | 4 | 2 | Mn | Schist | root |  |  |  |  |  |  |  |  |  |  |  |  | 3 | 0.01227 | ± | 0.00187 | 3 | 0.01442 | ± | 0.00283 |
| 213 | 9 | 4 | 3 | Mn | Schist | water |  |  |  |  | 3 | 0.02384 | ± | 0.00613 | 3 | 0.00328 | ± | 0.00044 | 3 | 0.00141 | ± | 0.00047 | 3 | 0.00217 | ± | 0.00090 |
| 214 | 9 | 4 | 4 | Mn | Schist | aa | 3 | 0.11356 | ± | 0.00262 | 3 | 0.08450 | ± | 0.03069 | 3 | 0.02310 | ± | 0.01361 | 2 | 0.01905 | ± | 0.01576 |  |  |  |  |
| 215 | 9 | 4 | 5 | Mn | Schist | ao | 3 | 0.34360 | ± | 0.01230 | 1 | 0.02387 |  |  |  |  |  |  |  |  |  |  |  |  |  |  |
| 216 | 9 | 4 | 6 | Mn | Schist | unextracted | 3 | 99.54284 | ± | 0.01443 | 3 | 99.88370 | ± | 0.04313 | 3 | 99.97362 | ± | 0.01392 | 3 | 99.95518 | ± | 0.01482 | 3 | 99.96368 | ± | 0.00682 |
| 217 | 10 | 1 | 1 | Fe | Basalt | shoot |  |  |  |  |  |  |  |  |  |  |  |  | 3 | 0.00003 | ± | 0.00001 | 3 | 0.00010 | ± | 0.00006 |
| 218 | 10 | 1 | 2 | Fe | Basalt | root |  |  |  |  |  |  |  |  |  |  |  |  | 3 | 0.00015 | ± | 0.00003 | 3 | 0.00023 | ± | 0.00004 |
| 219 | 10 | 1 | 3 | Fe | Basalt | water |  |  |  |  | 3 | 0.00001 | ± | 0.00000 | 3 | 0.00000 | ± | 0.00000 | 3 | 0.00001 | ± | 0.00000 | 3 | 0.00001 | ± | 0.00000 |
| 220 | 10 | 1 | 4 | Fe | Basalt | aa | 3 | 0.01490 | ± | 0.00030 | 3 | 0.01437 | ± | 0.00040 | 3 | 0.01649 | ± | 0.00019 | 3 | 0.01625 | ± | 0.00070 | 3 | 0.01630 | ± | 0.00068 |
| 221 | 10 | 1 | 5 | Fe | Basalt | ao | 3 | 5.93598 | ± | 0.03684 | 3 | 4.05949 | ± | 0.14949 | 3 | 4.06576 | ± | 0.45622 | 3 | 3.98648 | ± | 0.09946 | 3 | 4.40022 | ± | 0.41434 |
| 222 | 10 | 1 | 6 | Fe | Basalt | unextracted | 3 | 94.04912 | ± | 0.03671 | 3 | 95.92614 | ± | 0.14929 | 3 | 95.91775 | ± | 0.45604 | 3 | 95.99709 | ± | 0.10020 | 3 | 95.58313 | ± | 0.41376 |
| 223 | 10 | 2 | 1 | Fe | Rhyolite | shoot |  |  |  |  |  |  |  |  |  |  |  |  | 3 | 0.00013 | ± | 0.00005 | 3 | 0.00013 | ± | 0.00002 |
| 224 | 10 | 2 | 2 | Fe | Rhyolite | root |  |  |  |  |  |  |  |  |  |  |  |  | 3 | 0.00026 | ± | 0.00003 | 3 | 0.00038 | ± | 0.00007 |
| 225 | 10 | 2 | 3 | Fe | Rhyolite | water |  |  |  |  | 3 | 0.00004 | ± | 0.00001 | 3 | 0.00004 | ± | 0.00000 | 3 | 0.00007 | ± | 0.00002 | 3 | 0.00002 | ± | 0.00000 |
| 226 | 10 | 2 | 4 | Fe | Rhyolite | aa | 3 | 0.03075 | ± | 0.01792 | 3 | 0.03354 | ± | 0.00151 | 3 | 0.04591 | ± | 0.00335 | 3 | 0.03900 | ± | 0.00351 | 3 | 0.03076 | ± | 0.00200 |
| 227 | 10 | 2 | 5 | Fe | Rhyolite | ao | 3 | 1.95582 | ± | 0.09841 | 3 | 1.97391 | ± | 0.16405 | 3 | 1.86329 | ± | 0.14344 | 3 | 1.78132 | ± | 0.09922 | 3 | 1.90483 | ± | 0.10493 |
| 228 | 10 | 2 | 6 | Fe | Rhyolite | unextracted | 3 | 98.01343 | ± | 0.11459 | 3 | 97.99252 | ± | 0.16325 | 3 | 98.09076 | ± | 0.14037 | 3 | 98.17923 | ± | 0.09618 | 3 | 98.06388 | ± | 0.10328 |
| 229 | 10 | 3 | 1 | Fe | Granite | shoot |  |  |  |  |  |  |  |  |  |  |  |  | 3 | 0.00012 | ± | 0.00001 | 3 | 0.00009 | ± | 0.00003 |
| 230 | 10 | 3 | 2 | Fe | Granite | root |  |  |  |  |  |  |  |  |  |  |  |  | 3 | 0.00014 | ± | 0.00004 | 3 | 0.00021 | ± | 0.00004 |
| 231 | 10 | 3 | 3 | Fe | Granite | water |  |  |  |  | 3 | 0.00003 | ± | 0.00000 | 3 | 0.00002 | ± | 0.00000 | 3 | 0.00004 | ± | 0.00001 | 3 | 0.00004 | ± | 0.00001 |
| 232 | 10 | 3 | 4 | Fe | Granite | aa | 3 | 0.02562 | ± | 0.00123 | 3 | 0.02143 | ± | 0.00215 | 3 | 0.02210 | ± | 0.00191 | 3 | 0.01753 | ± | 0.00060 | 3 | 0.02100 | ± | 0.00143 |
| 233 | 10 | 3 | 5 | Fe | Granite | ao | 3 | 5.47833 | ± | 0.30748 | 3 | 4.30096 | ± | 0.66925 | 3 | 4.01667 | ± | 0.82253 | 3 | 5.24135 | ± | 0.40284 | 3 | 4.94663 | ± | 0.38957 |
| 234 | 10 | 3 | 6 | Fe | Granite | unextracted | 3 | 94.49606 | ± | 0.30841 | 3 | 95.67758 | ± | 0.67132 | 3 | 95.96120 | ± | 0.82413 | 3 | 94.74083 | ± | 0.40235 | 3 | 95.03203 | ± | 0.39063 |
| 235 | 10 | 4 | 1 | Fe | Schist | shoot |  |  |  |  |  |  |  |  |  |  |  |  | 3 | 0.00006 | ± | 0.00001 | 3 | 0.00007 | ± | 0.00002 |
| 236 | 10 | 4 | 2 | Fe | Schist | root |  |  |  |  |  |  |  |  |  |  |  |  | 3 | 0.00027 | ± | 0.00003 | 3 | 0.00027 | ± | 0.00003 |
| 237 | 10 | 4 | 3 | Fe | Schist | water |  |  |  |  | 3 | 0.00010 | ± | 0.00000 | 3 | 0.00007 | ± | 0.00000 | 3 | 0.00007 | ± | 0.00000 | 3 | 0.00010 | ± | 0.00001 |
| 238 | 10 | 4 | 4 | Fe | Schist | aa | 3 | 0.01429 | ± | 0.00060 | 3 | 0.00200 | ± | 0.00028 | 3 | 0.00187 | ± | 0.00026 | 3 | 0.00413 | ± | 0.00057 | 3 | 0.00284 | ± | 0.00048 |
| 239 | 10 | 4 | 5 | Fe | Schist | ao | 3 | 0.15184 | ± | 0.00667 | 3 | 0.06373 | ± | 0.01918 | 3 | 0.15232 | ± | 0.06306 | 3 | 0.15681 | ± | 0.01660 | 3 | 0.10039 | ± | 0.02560 |
| 240 | 10 | 4 | 6 | Fe | Schist | unextracted | 3 | 99.83386 | ± | 0.00712 | 3 | 99.93417 | ± | 0.01915 | 3 | 99.84574 | ± | 0.06301 | 3 | 99.83866 | ± | 0.01713 | 3 | 99.89634 | ± | 0.02611 |
|  |  |  |  |  |  |  |  |  |  |  |  |  |  |  |  |  |  |  |  |  |  |  |  |  |  |  |
